# The role of KDM5B in creating synthetic vulnerabilities in combination with radiotherapy in melanoma cells

**DOI:** 10.1186/s12964-026-02714-5

**Published:** 2026-03-05

**Authors:** Safa Larafa, Merle Schaffrin, Peer Braß, Renáta Váraljai, Sarah Scharfenberg, Nataly Kravchenko-Balasha, Gil Polinovski, Meenhard Herlyn, Nooraldeen Tarade, Stefan Wiemann, Alexander Roesch, Dirk Schadendorf, Verena Jendrossek, Johann Matschke, Batool Shannan

**Affiliations:** 1 Institute of Cell Biology (Cancer Research), Medical School, University Hospital Essen, University of Duisburg-Essen, Essen, Germany; 2 Department of Infectious Diseases, West German Centre of Infectious Diseases, University Duisburg-Essen, Essen, 45147 Germany; 3 Department of Dermatology, West German Cancer Center, University Hospital Essen, University Duisburg-Essen, Essen, Germany; 4 German Consortium for Translational Cancer Research (DKTK), Partner Site Essen/Düsseldorf, Essen/Düsseldorf, Germany; 5 The Institute of Biomedical and Oral Research, Hebrew University of Jerusalem, Jerusalem, 91120 Israel; 6 The Wistar Institute, Philadelphia, PA 19104 USA; 7 Division of Molecular Genome Analysis, German Cancer Research Center (DKFZ), Heidelberg, Germany; 8 West German Comprehensive Cancer Center Essen (CCC-WTZ)., Essen, Germany

**Keywords:** Intermittent cycling hypoxia, KDM5B, Tolerance, Synthetic vulnerability.

## Abstract

**Background:**

Cutaneous melanoma is the most aggressive type of skin cancer, with survival rates declining due to tumor heterogeneity and therapy resistance. Distinct subpopulations, including slow-cycling, therapy-resistant cells with high expression of the histone demethylase KDM5B, contribute to tumor progression and poor outcomes. Intermittent cycling hypoxia, defined by repeated hypoxia followed by reoxygenation, promotes tumor plasticity and aggressiveness, yet its role in melanoma heterogeneity and resistance remains poorly understood.

**Methods:**

We established hypoxia/reoxygenation-tolerant (HRT) melanoma cell lines (Hx10) through 10 cycles of intermittent cycling hypoxia (48 h at 0.2% O₂ followed by 120 h at 20.9% O₂) under conditions of KDM5B overexpression. Radiation response was evaluated in Hx10 and nonselected control cells. To investigate adaptive mechanisms, we performed reversed-phase protein array (RPPA) screening and applied an information-theoretic approach to compute protein-specific altered signaling signatures. Pathway enrichment analyses were used to identify dysregulated subnetworks.

**Results:**

Hx10 melanoma cells displayed increased resistance to radiation compared with nonselected control cells. Proteomic profiling identified distinct signaling signatures associated with KDM5B overexpression and adaptation to cycling hypoxia. These signatures revealed coexpressed subnetworks involving DNA repair, PI3K/AKT/mTOR, AMPK, and autophagy pathways, several of which are implicated in therapy resistance. Functional assays demonstrated that targeting either KDM5B or PI3K reduced the radioresistance of Hx10 melanoma cells. Sequential combination treatments impaired repopulation ability, particularly when KDM5B overexpression was withdrawn, indicating dependence on KDM5B for survival.

**Conclusions:**

Our findings provide proof-of-concept that altered signaling signatures can be used to define novel vulnerabilities in melanoma. KDM5B overexpression promotes adaptation to intermittent cycling hypoxia and confers resistance to radiation through activation of DNA repair and survival pathways. Targeting KDM5B or PI3K in combination with radiotherapy may represent a promising strategy to overcome resistance and improve treatment outcomes in melanoma.

**Supplementary Information:**

The online version contains supplementary material available at 10.1186/s12964-026-02714-5.

## Introduction

Tumor heterogeneity is a major hurdle in cancer treatment because different subpopulations of tumor cells may respond differently to therapies, leading to tumor recurrence and metastasis. Emerging evidence suggests that hypoxia is a major contributor to these challenges [1, 2].

Hypoxic regions tend to have a limited blood supply, thus making delivering anticancer therapies challenging. Moreover, hypoxia can drive several key processes that contribute to therapy resistance: [i] it rewires cellular metabolism by inducing a shift toward anaerobic glycolysis; [ii] it activates critical survival pathways, including hypoxia-inducing factor (HIF), enabling tumor cells to adapt to their microenvironment and evade therapy; and [iii] it promotes the presence of cancer stem cells and slow-cycling cells, both of which are associated with tumor recurrence, resistance to treatment, including radioresistance, and poor clinical outcomes [1, 3–5].

Hypoxia can occur in several forms, such as acute, chronic, or intermittent cycling hypoxia [6]. Evidence suggests that intermittent cycling hypoxia exerts unique selective pressure on tumor cells, favoring the survival of more aggressive subpopulations that are better equipped to withstand oxygen fluctuations, as demonstrated in cancers such as breast and lung cancers [7, 8]. These cells may exhibit specific adaptive features, including increased angiogenesis, altered metabolism (anaerobic glycolytic shift), or enhanced DNA repair mechanisms, allowing them to thrive under such conditions. Intermittent cycling hypoxia differs fundamentally from acute or chronic hypoxia because the repeated alternation between severe oxygen deprivation and reoxygenation imposes strong selective pressure, leading to robust metabolic and redox adaptation. In contrast to constant hypoxia, cycling hypoxia enhances ROS fluctuations, promotes activation of DNA repair programs, and induces stress-tolerant phenotypes [4, 9, 10]. Our previous work has shown that such hypoxia-reoxygenation dynamics drive resistance to ROS-inducing treatments, including radiotherapy, by stabilizing redox homeostasis and sustaining glutathione-dependent antioxidant pathways [7, 11, 12]. Complementary studies demonstrated that intermittent hypoxia induces apoptosis resistance, metabolic adaptation, and increased radiation survival [7, 11, 12]. Together, these findings highlight that cycling hypoxia creates a microenvironment that fosters aggressive, stress-resistant subpopulations and provides a biologically relevant context for interrogating KDM5B-driven adaptive mechanisms. While hypoxia is known to drive resistance to therapies (chemotherapy, radiotherapy, and immunotherapy), the exact mechanisms of interaction between hypoxia-induced pathways and drug resistance pathways remain incomplete. For example, how hypoxia synergizes with the MAPK/ERK or PI3K/AKT pathways to promote drug resistance has not been well studied. Moreover, hypoxia-induced epigenetic changes, such as histone modifications, play a role in therapy resistance, but their exact contribution to melanoma resistance remains to be elucidated [13–19]. As mentioned, intermittent cycling hypoxia is a form of hypoxia that leads to adaptation and treatment resistance [4, 5, 7, 8, 11], and it can increase the survival and growth of more aggressive tumor cells [7]. Previous work has revealed that intermittent cycling hypoxia can promote melanoma (i) growth via the secretion of proangiogenic factors, (ii) resistance, and (iii) EMT and immune system modulation [9]. However, this selective pressure can lead to dependency of these cells on certain pathways, presenting opportunities to exploit synthetic lethality or context-dependent vulnerability as a therapeutic approach.

Cutaneous melanoma is the most aggressive form of skin cancer, and survival rates dramatically decrease following distant organ metastasis. Despite advances in melanoma therapy, e.g., MAPK-targeted therapy (where ~ 70% of melanomas have activation of MAPK signaling, only approximately 60% with established druggable targets) and immune checkpoint blockade (e.g., anti-PD1 or anti-CTLA-4), metastatic melanoma remains difficult to treat owing to its intrinsic, adaptive, and acquired resistance mechanisms, as well as its extensive intratumoral heterogeneity [20–23]. Acquired therapy resistance is commonly associated with the emergence of therapy-resistant metastases in critical organs, such as the brain and bone, which often necessitate additional interventions such as radiation therapy (RT) [24]. However, resistance to RT can also develop, leading to rapid tumor relapse. This resistance is driven by the phenotypic plasticity of melanoma, including therapy-induced slow-cycling phenotypes and heterogeneity-associated markers such as KDM5B [25]. KDM5B (previously known as JARID1B) is a histone-3-lysine-4 (H3K4) histone demethylase that has a role in melanoma cell differentiation and cell state transitions [24, 26]. KDM5B was chosen as a central focus because it marks a slow-cycling, therapy-tolerant melanoma subpopulation and plays a functional role in multiple processes linked to resistance, including chromatin remodeling, metabolic adaptation, and DNA repair [24, 26–28]. Prior work has shown that KDM5B-high melanoma cells preferentially survive targeted therapy, display reversible phenotypic persistence, and re-enter proliferation after treatment withdrawal. More recent studies demonstrate that KDM5B regulates immune-evasive chromatin states and participates directly in DNA damage repair, distinguishing its role from other epigenetic regulators whose effects may be more context-dependent. Together, these data highlight KDM5B as a critical mediator of melanoma cell plasticity and a compelling mechanistic candidate for investigating adaptation to intermittent hypoxia and radiation.

Although melanoma has traditionally been considered radioresistant, radiotherapy remains an important treatment modality, especially for brain metastases and palliation. Several studies have demonstrated the benefit of combining RT with targeted therapy in melanoma brain metastases [29–31]. Modern studies show that up to 40–50% of melanoma patients develop brain metastases, where stereotactic radiosurgery (SRS) is a standard-of-care [32–34]. Historically, melanoma lesions demonstrated poor local control with conventional fractionation (83–90% local failure at 5 years) [35]. Hypofractionation and SRS have improved responses but melanoma still exhibits lower radiosensitivity compared to other solid tumors, partly due to high DNA repair capacity and hypoxia-associated resistance [36, 37]. Our recent findings indicated an important role of KDM5B in melanoma repopulation after combining targeted BRAF inhibition and RT. We also demonstrated that the degree of therapy resistance may even be dependent on the temporal treatment sequence chosen [24].

Melanoma’s ability to withstand recurrent environmental stressors aligns with previous evidence that tumor cells mount an early innate survival program that precedes the acquisition of stable drug resistance [38] and that hypoxia is a central driver of both cutaneous and uveal melanoma progression and therapy resistance [16]. Consistent with this biology, experimental models have demonstrated that hypoxia itself accelerates melanoma growth in vivo, such as in B16F10-bearing mice exposed to chronic intermittent hypoxia [9, 39], supporting the concept that oxygen fluctuations actively select for aggressive, stress-tolerant tumor phenotypes. The interactions between intermittent cycling hypoxia and tumor heterogeneity in melanoma remain poorly understood. Specifically, how these hypoxic conditions drive adaptive resistance mechanisms, enhance plasticity, and influence molecular pathways requires further investigation. Understanding these interactions could provide insight into the development of more effective therapeutic strategies that prevent or overcome treatment resistance.

To address these gaps, our study utilized reversed-phase protein array (RPPA) screening to profile melanoma cells exposed to intermittent cycling hypoxia with and without KDM5B overexpression. Through protein-specific signaling signature (PaSSS) analyses [40, 41] and STRING pathway enrichment, we identified synthetic vulnerabilities associated with these conditions. As a proof-of-principle, we evaluated the effects of combining sequential targeted therapy (e.g., PI3K/AKT inhibitors) with RT, focusing on the modulation of KDM5B and its role in driving melanoma repopulation posttreatment. These studies highlight the importance of integrating multilevel analyses to understand and target the adaptive responses of melanoma cells to hypoxia and therapy-induced stressors. Identifying key molecular players in these processes will be critical for overcoming resistance and improving patient outcomes.

## Materials & methods

### Cell culture

The human melanoma cell line was a kind gift from M. Herlyn (The Wistar Institute, Philadelphia, USA). In brief, WM3734 (Cellosaurus CVCL_6800) was established from human metastases of melanoma and is a BRAF^V600E^ mutant and NRAS wild type. For all experiments, hypoxia-adapted (Hx10) and control (Ctl) WM3734 cells were cultured in the same melanoma medium with identical FBS concentration (2% fetal bovine serum (FBS)-substituted melanoma medium (Tu2%) as previously described [42] and grown at 37 °C in 5% CO2), seeding densities, medium change schedule and incubation conditions (37 °C, 5% CO₂). The only experimental variable that differed between groups was oxygen tension: Hx10 cells were subjected to 10 cycles of severe hypoxia (0.2% O₂, 48 h) followed by reoxygenation (20.9% O₂, 120 h), whereas Ctl cells were maintained continuously under normoxic conditions without oxygen cycling. The cells were irradiated with X-ray ionizing radiation at doses ranging from 2 to 10 Gy. Photons were created with an X-RAD 320 X-ray Biological Irradiator with an MIR-324 tube and a 1.65 mm beam conditioning filter. The machine has an initial energy of 320 keV and a dose rate of 3.40 Gy/min. The cells were radiated at a distance of 50 cm from the X-ray tube window. The cells were returned to the incubator immediately after exposure to ionizing radiation. The MAPKi inhibitors GSK458 and MK2206 (Cat # S2658 and # S1078, respectively) were purchased from Selleckchem (Munich, Germany). All the compounds were stored at -20 °C in dimethyl sulfoxide (DMSO) as 10 mM stocks. Doxycycline (dox)-inducible KDM5B overexpression was performed as previously described [27]. For combination sequential treatment, the sequence applied was MAPK inhibition [either GSK458 (PI3Ki) or MK2206 (AKTi) (TT)] followed by irradiation (IR). In brief, once WM3734 melanoma cells reached ~ 70% confluence, the cells were treated with MAPK inhibitors on day 0 and then irradiated on day 3. The cell numbers used for the assays and the temporal sequences were designed in such a way that cell density effects could be excluded. Dox addition [10 ng/mL] caused KDM5B overexpression (OE) in WM3734^KDM5B^ melanoma cell lines. All the cell lines were authenticated via STR profiling and regularly tested for potential mycoplasma contamination.

siRNA KDM5B, siRNA Ctl (Qiagen) and siRNA p110α (Cell Signaling) were used according to the manufacturer’s instructions.

#### Limiting dilution assay (LDA):

Melanoma cells were counted, diluted to 1,000, 500, 250, 125, 62.5, 31.25, 15.6, and 7.8 cells/well and seeded into 96-well plates (6 replicates/condition). The cells were treated with GSK458 (0.01 or 0.05 µM) for 24 h and then irradiated with a single dose (0, 2, or 5 Gy) for 24 h. The cells were cultured for 10‒30 days, fixed (3.7% PFA), stained (comprising blue) and counted. Each well was counted for clonogenic growth. For the analysis, those values were copied and added to the ELDA website for analysis (https://bioinf.wehi.edu.au/software/elda/). The software assumed that 0 Gy of DMSO would result in 100% cell survival. The survival fraction was then calculated.

#### Cell cycle analysis:

Melanoma cells were seeded into 6-well plates at a density of 300,000 cells per well and incubated at 37 °C (5% CO₂) for 24 h. After incubation, the culture medium was removed, and the cells were washed with PBS. The cells were then trypsinized, and PBS was added to neutralize the trypsin. The cell suspension was collected and centrifuged at 1500 rpm for 10 min. The supernatant was discarded, and the cell pellet was resuspended in 100 µL of fixation/permeabilization buffer (FOXP3/transcription factor staining buffer set; Invitrogen, Cat: 00–5523–00) and incubated for 30 min at room temperature. Subsequently, 200 µL of permeabilization buffer (FOXP3 Kit) was added, and the mixture was centrifuged at 1500 rpm for 10 min. Following removal of the supernatant, the cells were stained with DAPI (1:2500 dilution in permeabilization buffer) for 1 h at room temperature in the dark. Analysis was performed via flow cytometry (Beckman Coulter cytometer).

### Proliferation and colony formation assays

Cell numbers and viability were assessed via standard protocols involving crystal violet (CV) staining. The procedure involved seeding the cells in 24-well plates and allowing them to proliferate to approximately 70% confluence. For crystal violet staining, the cell culture medium was removed, and the cells were washed and fixed with 70% ethanol for 1 h at room temperature. Then, the cells were stained with 1% crystal violet for 30 min, washed, and dried. The stained cells were photographed, and the dye was dissolved in 70% ethanol for measurement of the emission at 550 nm via an Epoch Microplate Spectrometer (Bio-Tek, Winooski, VT, USA). Image analysis was performed via ImageJ-win64 software. Three independent experiments were conducted, and the standard deviation (SD) was calculated.

For cell cycle analysis, propidium iodide (PI) staining was performed. Melanoma cells were seeded in 6-cm plates and allowed to reach approximately 70% confluence. The cells were subsequently irradiated with different doses (0, 5, or 10 Gy). After 1 or 3 days, the cells were trypsinized, washed with PBS containing 5 mM EDTA, and incubated with 100% ethanol. RNase A was added, followed by PI at a final concentration of 100 µg/mL. The intercalation of PI into chromosomal DNA was measured via a Gallios cytometer (Beckman Coulter), and data analysis was performed via FlowJo software (version 7.6.5).

For colony formation assays (CFAs), clonogenic cell survival in response to the different treatments was determined by comparing the clonogenic survival of Ctl (normoxic) cells and Hx 10 (anorexia/reoxygenation-tolerant) hypoxia cells. In brief, the cells were irradiated (0 to 5 Gy) and plated in agar on 6-well plates at a density of 5,000 cells per well. The plates were subsequently incubated for ~ 3 weeks before quantification of colony formation. For this purpose, the cells were fixed in 3.7% formaldehyde and 70% ethanol and stained with 0.05% Coomassie blue, and colonies of at least 50 cells were counted by GelCount (Oxford Optronix, Oxfordshire, Great Britain). The plating efficiency and surviving fraction (SF) under the corresponding Ctl and HRT hypoxia conditions were calculated as described elsewhere [12]. 

### Assessment of cell viability via 7-AAD staining

To evaluate the impact of IR on cell death, melanoma cells were stained with 7-AAD and analyzed by flow cytometry. The cells were seeded in 6-well plates (300,000 cells/well) and incubated at 37 °C (5%CO_2_) for 24 h. The cells were then treated with different IR doses (2 Gy, or 5 Gy) or left untreated for the control groups. After 24 h, 48 h and 72 h, the cells were washed 1x with PBS, detached with accutase (the supernatants were collected in the same tubes), and centrifuged for 5 min at 1500 rpm. The supernatant was discarded, and the pellet was stained with 7-AAD (1:1000) in PBS for 30 min at 37 °C. Cell death was analyzed by flow cytometry (Beckman Coulter cytometer), and > 10,000 events were analyzed in single cells via the PC5.5 channel (excitation: 488, emission: 699). The analysis was performed via CytExpert software. 

### Assessment of cell viability via trypan blue staining

For the Trypan blue viability assay, 50,000 human melanoma cells were seeded into 6-well plates and subjected to the indicated treatments as described in the corresponding figure legends. At the specified time points, both adherent and floating cells were collected and resuspended, and 10 µL of cell suspension was mixed with 10 µL of Trypan blue solution. Viable (unstained) and non-viable (blue-stained) cells were counted using a Neubauer hemocytometer.

### Western blot

Proteins were extracted as described previously [42], and 20 mg of cell extract was separated on a 10% polyacrylamide‒sodium dodecyl sulfate gel before being transferred onto a nitrocellulose membrane (Roth, Karlsruhe, Germany). Primary antibodies were incubated at 4 °C overnight in Tris-buffered saline containing 0.1% Tween-20 (TBS-T) and 5% milk. The primary antibodies used were rabbit anti-KDM5B 1:1200 (NB100-97821) and tubulin (Cell Signaling, Cat# 2148). The blots were subsequently washed with TBST and incubated for 1 h with either anti-rabbit (115–035–046) or anti-mouse (115–035–003) horseradish peroxidase-conjugated secondary antibodies (Jackson ImmunoResearch Laboratories Inc., Missouri, USA) diluted 1:10 000 in TBS-T and 5% milk. Western blots were visualized via an enhanced chemiluminescence system (WesternBright Chemiluminescence Substrate, Biozym, Hessisch Oldendorf, Germany). Then, the western blots were scanned via the LAS300 Imaging System (Fuji, Munich, Germany). For quantification, the images were analyzed using ZEN software version 2.3 lite.

### Collagen-embedded melanoma spheroids

#### Cell invasion assay

Melanoma spheroids were generated as previously described [24, 25]. Briefly, 2,500 cells were seeded on 1.0% agarose-coated, non-adherent 96-well plates to allow spheroid formation. After 72 h, intact spheroids were embedded in a collagen-based matrix to assess three-dimensional (3D) invasion. Embedded spheroids were treated with the indicated compounds, with specific treatment conditions and time points detailed in the corresponding figure legends. Spheroid invasion was monitored by image acquisition using a Zeiss AxioObserver.Z1 inverted microscope, and images were analyzed with ZEN software (version 2.3 lite).

#### Live /Dead staining

Cell viability within melanoma spheroids was assessed using the LIVE/DEAD^®^ Viability/Cytotoxicity Kit according to the manufacturer’s instructions (Invitrogen™, Waltham, MA, USA). Melanoma spheroids were generated as previously described [42]. Prior to staining, spheroids were washed with PBS and incubated with the combined LIVE/DEAD^®^ reagents diluted in PBS (v/v). Fluorescence signals were visualized using a Zeiss AxioObserver.Z1 microscope, and images were processed using ZEN software (version 2.3 lite).

#### Reverse-phase protein arrays

Targeted RPPA experiments were performed as previously described [43]. Briefly, cell lysates from biological replicates for each condition were spotted on nitrocellulose-coated glass slides (Oncyte Avid, Grace-Biolabs, Bend, OR, USA) in technical triplicates via an Aushon 2470 contact printer with 185 μm solid pins. Primary antibodies were previously validated via Western blotting to validate their specificity. The signal intensities of the spots were quantified via GenePixPro 7.0 (Molecular Devices). RPPA raw data preprocessing and quality control were carried out via RPPanalyzer [44]. The intensity values were log2 transformed and plotted via Morpheus software (https://software.broadinstitute.org/morpheus).

The untargeted RPPA assays and data processing were conducted at the MD Anderson Center RPPA core facility in Houston, TX, USA (refer to bib33 and https://www.mdanderson.org/research/research-resources/core-facilities/functional-proteomics-rppa-core/rppa-process.html). The resulting readouts, represented on a logarithmic scale (log_2_), were normalized via median centering across the antibodies. To analyze the normalized log_2_ median-centered protein values, unsupervised hierarchical clustering was performed via Cluster 3.0 software, which employs centered correlation and complete linkage methods (http://bonsai.hgc.jp/~mdehoon/software/cluster/software.htm#ctv).

#### Surprisal Analysis

In this study, a thermodynamic-based information-theoretic approach called surprisal analysis was utilized (as described previously [40, 45]). The analysis is based on the concept that biological systems reach a state of equilibrium in the absence of constraints [40]. However, when influenced by environmental and genomic constraints, the system deviates from its state of minimal free energy. These constraints can induce specific changes in the protein network of cells, forming what is termed an “unbalanced process.” Multiple constraints can impact the system, leading to the emergence of several unbalanced processes. In characterizing tumor systems, the specific set of unbalanced processes constitutes the tumor-specific signaling signature and is known as the protein-specific signaling signature (PaSSS).

Surprisal analysis aims to identify the complete set of constraints operating on the system in a given tumor, denoted as (k). It employs the following equation: ln Xi(k) = ln Xi0(k) - ΣGiαλα(k). Here, (Xi) represents the expression level of the protein of interest, (Xi0) denotes the expected expression level when the system is at a steady state without constraints, and ΣGiαλα(k) represents the sum of deviations in the expression level of protein i due to various constraints.

The term Giα represents the involvement of protein i in the unbalanced process α. Proteins exhibiting significant Giα values are grouped into active unbalanced processes observed in the dataset. The negative/positive amplitude indicates the correlation of cells with respect to a specific process. Each process is indexed as α = 1, 2, 3, etc., with decreasing significance as the index process increases. For example, unbalanced process 1 is present in more cells than unbalanced processes 2 and 3 are. While multiple unbalanced processes may exist within a system, not all processes are active in all cells. The sum Σα = 1Giαλα(cell) represents the information available for each protein. The deviations in the expression level of proteins caused by different constraints contribute to the overall change in the expression level. A protein influenced by constraints associated with one or more unbalanced processes cannot adopt any arbitrary expression level. Its expression level is impacted by the expression levels of other proteins within the unbalanced process occurring in the cell. In this study, we analyzed protein expression data obtained from RPPA analyses.

#### Immunofluorescence staining of yH2AX:

Melanoma cells were seeded onto coverslips placed in 12-well plates at a density of 100,000 cells per well and incubated at 37 °C (5% CO₂) for 24 h. Following ionizing radiation (IR) treatment, the culture medium was removed, and the cells were washed with PBS. The cells were fixed in 1 mL of 3% paraformaldehyde (PFA) mixed with Triton X-100 and subsequently blocked in 2% normal goat serum (NGS) solution at 4 °C overnight. The coverslips were then incubated with an Alexa Fluor 647-conjugated mouse anti-γH2AX (pS139) antibody (BD Pharmingen, Cat: 560447) diluted 1:100 in NGS solution (50 µL per coverslip) for 1 h in the dark. After two washes with PBS, the cells were counterstained with Hoechst for 15 min in the dark, followed by 3 additional washes with PBS. Coverslips were mounted onto slides using DAKO mounting medium and allowed to dry at 4 °C for 48 h in the dark. Microscopy images of at least 50 foci per sample were captured, and γH2AX foci were quantified via Focinator software.

#### Statistics:

Unpaired *t* tests (Student’s *t* tests) were used to compare mean differences between two independent groups. One-way unpaired *t* tests were performed in GraphPad Prism (versions 6–8), which were conducted at the two-sided significance level, where *p* values of ≤ 0.05 were considered significant. Ordinary one-way ANOVA was performed for the analyses of the LDA (Supplementary Fig. 5).

## Results

### KDM5B impacts adaptation to cycling hypoxia/reoxygenation stress and facilitates the selection of melanoma cells with associated radiation resistance

**Fig. 1:**
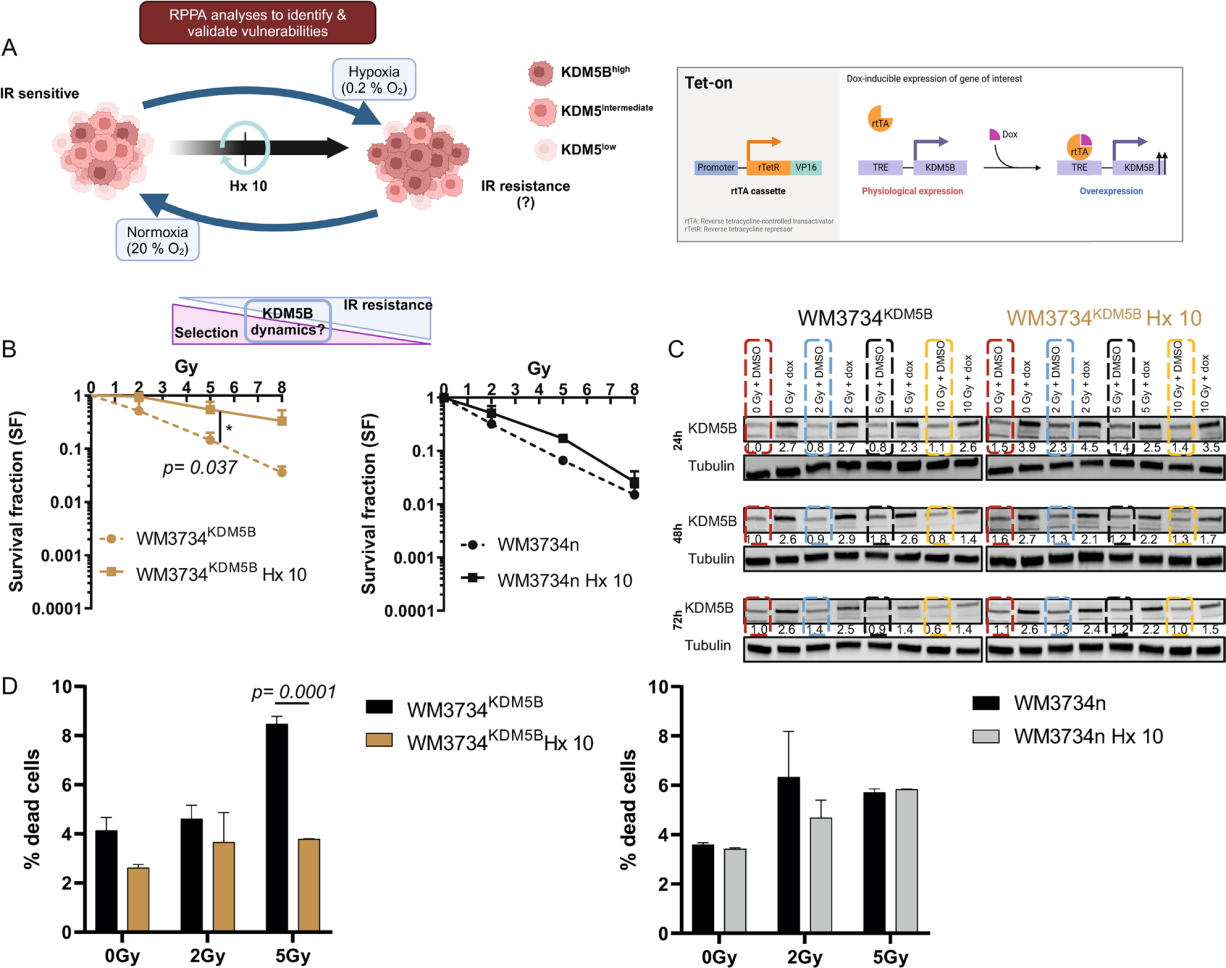
Cycling hypoxia creates vulnerability to IR. **A** Schematic diagram depicting the process of generating cell lines via intermittent cycling hypoxia. Relatively RT-sensitive melanoma cell lines (WM3734^KDM5B^ and WM3734^n^) were exposed to repetitive cycles of cycling hypoxia for 10 weeks (Hx 10) (Table 1) to overexpress KDM5B. The resulting cell lines (Ctl and Hx 10) were analyzed for their phenotype and protein expression via RPPA. **B** Schematic diagram of the KDM5B-Dox inducible system. A and B were generated via BioRender. **C** Survival fraction following a colony formation assay (CFA) of Ctl and Hx10 hypoxic cell lines following increasing doses of IR for 48 h. **D** Percentage of dead cells following increasing doses of IR for 48 h. The bar graphs present the means ± SDs from *n* = 2 independent experiments. Significance was tested via Student’s t test

In unstressed situations, melanomas exhibit heterogeneous expression of KDM5B, with high, intermediate and low expression [27]. Stress conditions (e.g., hypoxia) lead melanoma cells to upregulate KDM5B expression as a survival mechanism. These dynamics of KDM5B expression are vital for identifying mechanisms to understand hypoxia-mediated resistance and eliminate melanoma cells (Fig. 1A- left). To investigate cellular adaptation to cycling hypoxia, its potential contribution to radiation resistance, and the role of KDM5B in this process, we established an experimental model by generating hypoxia/reoxygenation-tolerant (HRT) WM3734^n^ melanoma cells through repeated exposure to 10 cycles of severe hypoxia (0.2% O₂ for 48 h) followed by reoxygenation (120 h at 20% O₂) (Fig. 1A- left).

To specifically focus on the role of KDM5B in this adaptation process, we also generated a KDM5B-overexpressing (OE) cell system (WM3734^KDM5B^) from the naïve WM3734 (WM3734^n^) melanoma cell line (Fig. 1A-right). WM3734^KDM5B^ melanoma cells contain a doxycycline-inducible construct for KDM5B (WM3734^KDM5B^, previously described [27]), where the addition of doxycycline (dox) induces KDM5B OE (Fig. 1A-right). These cells were generated from KDM5B-overexpressing (+ dox) WM3734^KDM5B^. In addition, we generated a control cell line, which was maintained under normoxic conditions (Ctl^n^ from WM3734^n^ and Ctl^KDM5B^ from WM3734^KDM5B^).

In total, we generated 4 cell lines: WM3734^KDM5B^ (Ctl ^KDM5B^, Hx 10 ^KDM5B^), in which KDM5B was overexpressed (+ dox), and WM3734^n^ (Ctl^n^, Hx 10^n^), in which endogenous KDM5B was expressed. For our experiments, we utilized either the two WM3734^n^ cell lines (Ctl, Hx 10) with endogenous KDM5B expression or the two WM37347^KDM5B^ cell lines (Ctl, Hx 10) in the presence or absence of dox (KDM5B overexpression) to elaborate on the role of KDM5B in the adaptation process. In some experiments, we seeded WM3734^KDM5B^ (Ctl, Hx 10) in the presence of dox and then removed it to assess how the loss of OE KDM5B affects survival. This modulation of KDM5B is important for modeling any changes that melanoma cells undergo when stressed. The different cell lines are summarized in Table 1.

After our working models were established, we investigated [i] whether the selection process had an effect on cellular radiosensitivity and [ii] whether KDM5B overexpression during the selection process had an effect on the resulting adaptive processes, and we performed standard long-term colony formation assays in all the cell lines (Fig. 1B).

**Table 1:** Description of the cell lines used and where they appear in the figure

Cell lines	Description	Results in figures
WM3734^KDM5B^ (Ctl, Hx 10) without dox	The cells were generated under dox (KDM5B OE), **however**,** cells were seeded without dox** to assess if the resulting KDM5B-related phenotypes were transient or permanent	1B-D, S1A-C (left), 2D/E (-dox), 4, S4E, 5, 6
WM3734^KDM5B^+ dox (Ctl, Hx 10)	The addition of dox induces KDM5B OE. WM3734^KDM5B^ (Ctl, Hx 10) cells were generated under dox- see Fig. 1	1C, 2, 3, S3, 4, 5, 6
WM3734^n^ (Ctl, Hx 10)	Endogenous KDM5B expression	1B-D, S1A-C (right), S2, S4, S5, 6B (right)
Ctl	Cells were kept in normoxic conditions	1B-D, S1A-C (right), S2, S4, S5, 6B (right)
Hx 10	Cells were exposed to hypoxia-reoxygenation for 10 cycles	1B-D, S1A-C (right), S2, S4, S5, 6B (right)

Investigation of cellular radiosensitivity via colony formation assays revealed a significantly greater survival fraction (SF) in Hx 10^KDM5B^ cells than in control^KDM5B^ cells (*p* = 0.037) in the absence of dox. These effects were not significantly different in WM3734^n^ Hx 10 cells, although Hx 10^n^ cells presented increased survival at lower radiation doses than Ctl^n^ cells did. These results indicate that enhanced radioresistance following adaptation to intermittent cycling hypoxia in WM3734^KDM5B^ Hx 10 cells is likely due to the influence of KDM5B overexpression during Hx 10 cell adaptation to chronic hypoxia stress (Fig. 1B- left).

Next, cell cycle/PI analysis (0, 2, and 5 Gy) of cycling hypoxia-tolerant WM3734^KDM5B^ Hx 10 and Ctl cells in the absence of dox did not reveal significant differences; however, there was a relative increase in the percentage of Hx 10KDM5B cells in the S phase compared with that of Ctl^KDM5B^ cells (Supplementary Figure S1A- left). These data indicate that WM3734 Hx 10^KDM5B^ cells acquired a more proliferative and adaptive phenotype in response to irradiation. On the other hand, compared with Ctl cells, WM3734^n^ Hx 10 cells exhibited a strong decrease in the number of cells in the S phase (Supplementary Figure S1A-right). We hypothesized that the effects observed in hypoxia-tolerant WM3734^KDM5B^ Hx 10 cells may result from their generation under conditions of KDM5B OE. To confirm this, we knocked down KDM5B expression via siRNA. Cell cycle/PI analysis revealed a relative increase in G2/M following KDM5D KD, indicating a partial protective effect of KDM5B following ionizing radiation (IR) (Supplementary Figure S1B).

We then assessed KDM5B expression at the protein level following exposure to various radiation doses and monitored its dynamics over time. KDM5B expression was greater in Hx 10^KDM5B^-treated cells than in control cells at 0, 2, 5, and 10 Gy (red, blue and yellow dotted lines, respectively) and in Ctl^KDM5B^-treated cells than in control cells (Fig. 1C). The elevated KDM5B expression in the Hx 10^KDM5B^ cell line may have contributed to the enhanced radioresistance observed in the long-term colony formation assay (Fig. 1B). This effect was not consistently observed in WM3734^n^ melanoma cells (Supplementary Fig.  S1 C). Dox addition increased the expression of KDM5B in WM3734^KDM5B^ but not in WM3734^n^ melanoma cell lines, confirming the functionality of our system. Our findings suggest that intermittent cycling hypoxia results in the selection of melanoma cells with tolerance to hypoxia/reoxygenation and increased resistance to IR, with adaptation being facilitated by KDM5B overexpression.

To investigate whether the differences in radiosensitivity between Hx10 and Ctl cells can be attributed to differences in response to IR, e.g., acute cell death, we assessed cell death upon acute exposure to different doses of IR (24 h). In fact, acute cell death, as measured by flow cytometry of cells stained with 7AAD, was more prominent in WM3734^KDM5B^ Ctl cells than in Hx 10 cells (Fig. 1D- left). Differences were less prominent when cells were irradiated with lower radiation doses in WM3734^KDM5B^ and in the WM3734^n^ cells (Fig. 1D).

Our data indicate that KDM5B impacts the adaptive capabilities of melanoma cells to hypoxia/reoxygenation stress, with relevance to the acute radiation response and radiosensitivity. These findings also indicate that intermittent cycling hypoxia creates hypoxia-tolerant and RT-resistant melanoma cell lines that are more prominent in the presence of KDM5B (WM3734^KDM5B^ with or without dox).

### Proteomic analyses revealed the modulation of key survival signaling pathways

**Fig. 2:**
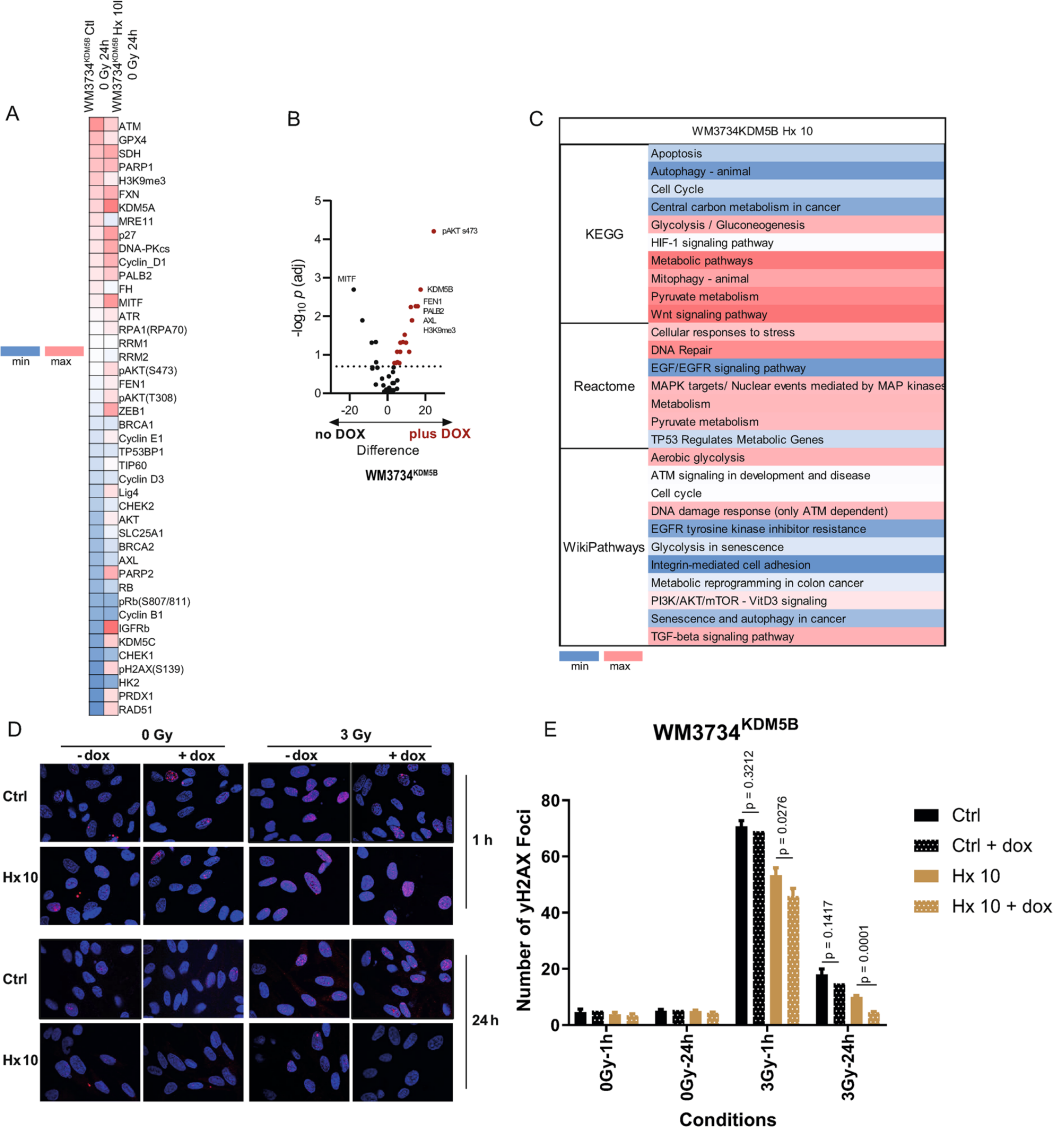
RPPA analyses revealed the modulation of survival signaling pathways. **A** Heatmap showing different protein expression levels (log_2_ normalized) between the WM3734^KDM5B^ + dox Ctl and Hx 10 cell lines following the targeted RPPA. **B** Volcano plots visualizing the differences in protein expression (log_2_-fold change) and q values (-log_10_ adjusted p values) in WM3437^KDM5B^ with and without dox addition. **C** STRING pathway analyses revealed an increase in survival pathways, including autophagy, DNA repair, metabolism, and glycolysis, in WM3734^KDM5B^ Hx 10 cells compared with those in Ctl cells. Enrichment scores (-log_10_ adjusted p values) of functional pathways as defined by the Wiki, Reactome, and KEGG functional pathway databases and GO biological processes. **D** Gamma H2AX foci staining following IR in WM3734^KDM5B^ melanoma cell lines with and without dox addition. **E** Quantification of Gamma H_2_AX foci following IR in WM3734^KDM5B^ melanoma cell lines. Significance was tested via Student’s t test. The experiments were repeated *n* = 3 times unless otherwise mentioned

To gain more insight into the impact of the selection process in the presence or absence of KDM5B overexpression on the cellular phenotype and associated differences in radiosensitivity, we functionally analyzed the different cell lines described in Table 1 at the protein level via RPPA screening. Therefore, we performed *targeted RPPA profiling* (45 proteins) and *untargeted RPPA profiling* (490 proteins) to analyze changes in well-described radiation- and melanoma biology-related pathways upon IR treatment. In the targeted RPPA, comparison between baseline (0 Gy) Hx 10 and Ctl WM3734^KDM5B^ cell lines under KDM5B overexpression (*in the presence of dox* for 24 h) revealed an increase in the DNA damage response and repair (ATR, FEN1, PARP2, H2AX, RAD51, FH, and KDM5C), cell survival, growth, and stress response (AKT, pAKT, IGFRb, Cyclin E1, ZEB1, and PRDX), and epigenetic regulation (H3K9me3, KDM5C, and H2AX). These proteins are critical nodes in the DNA damage response, chromatin regulation, and survival signaling pathways. Together, they define how cells balance genome stability with stress adaptation, which is especially relevant in cancer progression and therapy resistance (Fig. 2A). We visualized the data in a volcano plot, which displayed the upregulated proteins (Fig. 2B). STRING analyses revealed increases in the metabolic, MAPK/PI3K and DNA damage response pathways (Fig. 2C).

To confirm our results, we performed a similar comparison in WM3734^n^ (Hx 10 vs. Ctl). The RPPA results revealed that these cells are repair compromised but stress adapted, which makes them hypersensitive to DNA-damaging agents and more dependent on compensatory pathways (FEN1, PRDX1, RRM2) (Supplementary Fig.  2 A). Since WM3734^n^ cells do not contain a KDM5B overexpression construct, we used a chemical compound, Cpd1, to induce KDM5B. Cpd1 is a tool compound that indirectly increases the expression of KDM5B (described in [27]).

We visualized the data following treatment with Cpd1, which induced KDM5B expression, via a volcano plot [27] (Supplementary Fig. 2B). These data support the role of KDM5B in mediating RT resistance in melanoma cells.

### The role of KDM5B in mediating radiation resistance in Hx 10 HRT melanoma cells

In our previous work, we identified KDM5B as a therapy overarching resistance marker that facilitates cross-resistance in melanoma [24, 27]. Our current results revealed that KDM5B OE leads to increased cell survival after IR (Fig. 1). Untargeted RPA analyses revealed an increase in DNA repair pathways, so we performed immunofluorescence imaging for gamma (γ) H2AX in WM3734^KDM5B^ cells in the presence or absence of dox treatment. Compared with the control, the Hx 10 WM3734^KDM5B^ melanoma cell line presented the least amount of DNA damage (red puncta) following irradiation at a dose of 3 Gy *in the presence of dox* (+ dox) (Fig. 2D, E). Importantly, the removal of dox (- dox) led to a significantly greater number of residual γH2AX foci in Hx 10 (*p* = 0.0001, and *p* = 0.0276 compared with dox and no dox only in Hx 10 WM3734^KDM5B^ melanoma cells after 24 h, 3 Gy and 1 h, 3 Gy, respectively), but not in Ctl WM3734^KDM5B^ melanoma cells (*p* = 0.3212, and *p* = 0.1417 compared with dox and no dox in Ctl after 24, 3 Gy and 1 h, 3 Gy, respectively) (Fig. 2E). These results highlight the importance of KDM5B in the development of hypoxia/reoxygenation tolerance by potentially increasing DNA damage repair. The involvement of KDM5B in DNA repair has been previously shown in lung cancer cells [28]. In the WM3734^n^ cell line, a similar trend was observed as that in the WM3734^KDM5B^ cell line, where WM3734^n^ Hx 10 cells presented significantly fewer γH2AX foci after 0 Gy for 1 h (*p* = 0.011) and after 3 Gy for 24 h (*p* = 0.0447) (Supplementary Fig.  2 C, D). Our findings suggest that KDM5B contributes to the development of cellular tolerance to ionizing radiation.

Untargeted RPPA analyses revealed that KDM5B overexpression during the adaptation process to cycling hypoxia increases the activity of the MAPK and PI3K signaling pathways as survival mechanisms

**Fig. 3:**
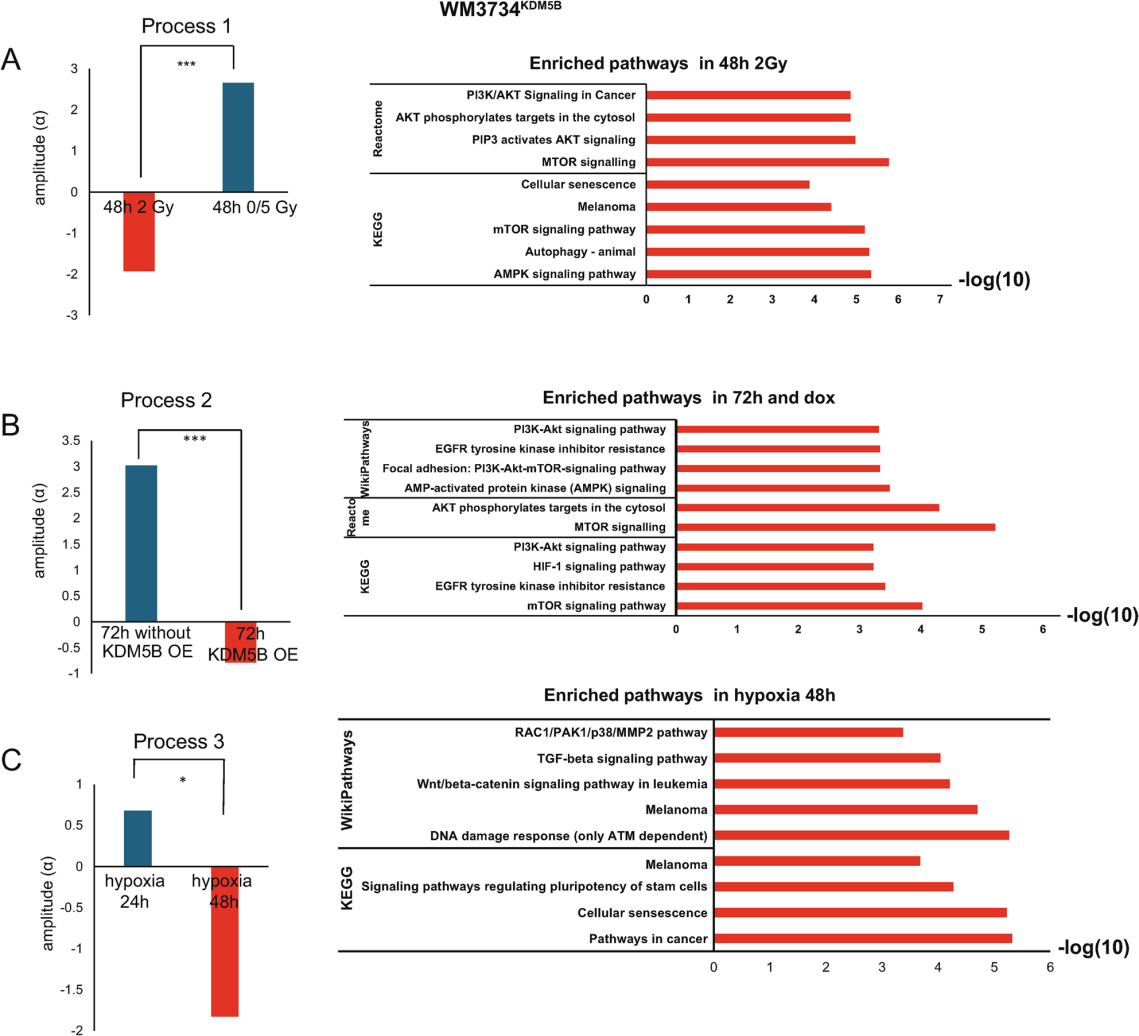
RPPA analyses revealed that KDM5B overexpression increases MAPK and PI3K signaling. PaSSS (protein-specific signaling signature) and STRING pathway enrichment analyses of protein expression level changes in different imbalance processes. The analyses revealed a subset of proteins enriched at 48 h in the 0 and 5 Gy groups (unbalanced process 1–**A**), those enriched in the KDM5B OE group after 72 h (unbalanced process 2–**B**), and those enriched in the Hx 10 group (unbalanced process 3–**C**). STRING analyses are on the right-hand side

To identify protein pathways that could explain the increased survival of Hx 10^KDM5B^ cells compared with that of Ctl^KDM5B^ cells following IR, we performed expanded untargeted RPPA analyses in collaboration with the Lab of Meenhard Herlyn (Wistar Institute, Philadelphia, USA), which covers 490 proteins. WM3734^KDM5B^ melanoma cells were then irradiated (0, 2–5 Gy), and lysates were collected 24, 48, or 72 h after IR (Fig. 3). The expanded untargeted RPPA analysis was followed by protein-specific signaling signature (PaSSS) and STRING analyses (previously described [40, 41]).

**Fig. 4:**
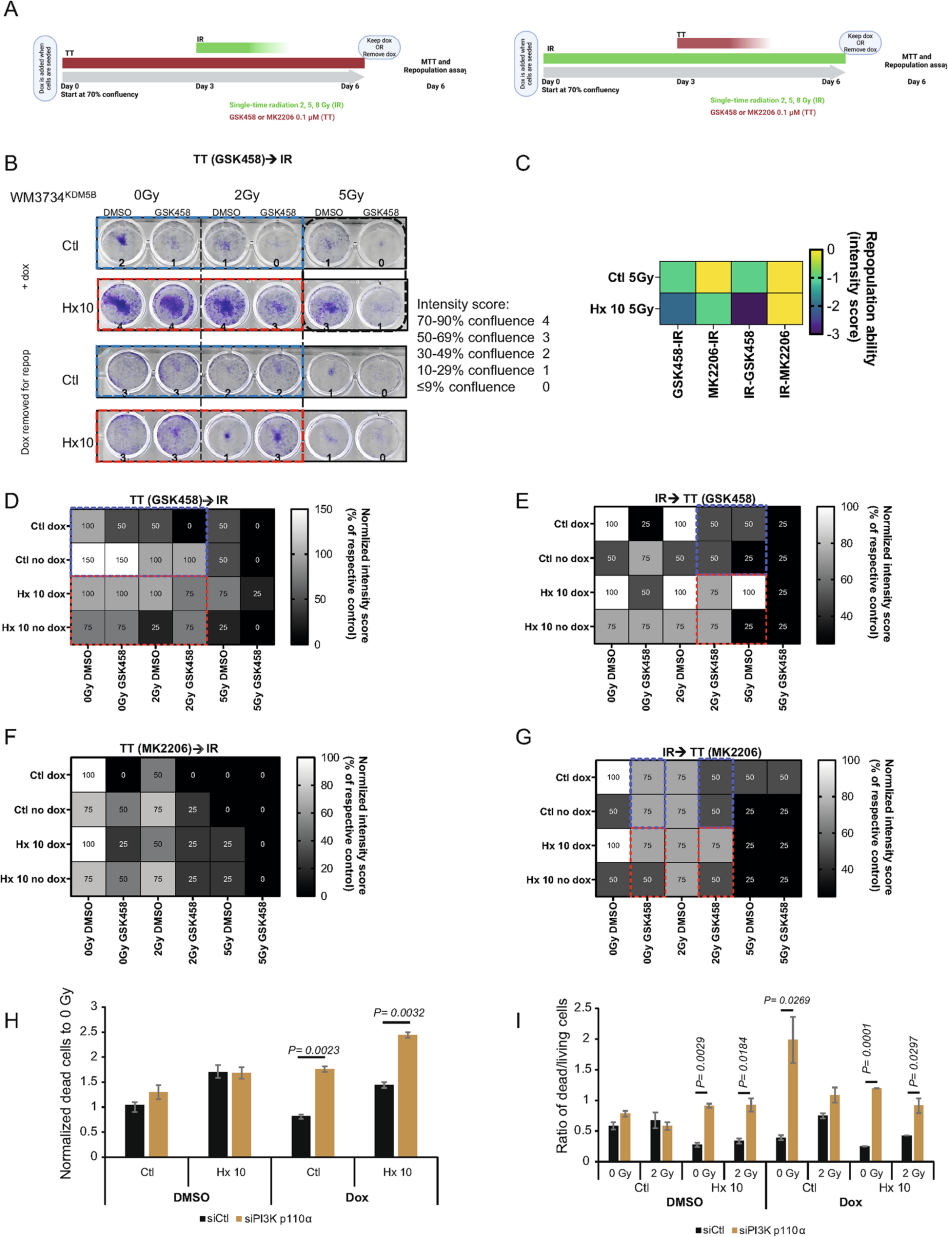
Different sequential treatments affect the repopulation of melanoma cell lines. **A** Schematic diagram of the treatment sequences. **B** Representative example of a repopulation assay. **C** Heatmap of the repopulation ability intensity score (black box in **B**), indicating that Hx 10 cells have reduced repopulation abilities compared with Ctl cells. **D**—**E** Heatmaps of normalized intensity scores of repopulation abilities following sequential combination treatment (TT→IR and IR→TT) in WM3734^KDM5B^ melanoma cell lines. GSK458 and MK2206 were used at 0.01 µM in WM3734^KDM5B^ melanoma cell lines (Ctl, and Hx 10) with dox. The cells were treated with sequential combination therapy (TT→IR and IR→TT). GSK458 or MK2206 (0.1 µM) was given for 3 days, after which the cells were treated with 0–5 Gy of IR for another 3 days (and vice versa), after which a repopulation assay was performed. The repopulation assay was stopped when at least one well reached ~ 100% confluence. The blue and red boxes indicate points of comparison. The intensity score reflects the degree of well confluence. **H** and **I** Trypan blue staining of dead cells following 72 h siRNA p110α or siCtl and 72 h IR (0, 2 Gy) with or without dox treatment (KDM5B OE). Results are depicted as a percentage of dead cells (**H**), or a ratio of dead to living cells (**I**). Significance was tested via Student’s t test

PaSSS analysis revealed 3 unbalanced processes/networks in WM3734^KDM5B^ Ctl and Hx 10 cells [40, 46]. These processes appeared in at least 40% of the samples, with each describing a different experimental condition. The first network was the most abundant, appearing in 80% of the samples, whereas the third network was the least abundant. Specifically, in WM3734^KDM5B^ dox-treated cells (i.e., overexpressing KDM5B), proteins associated with imbalanced processes 1, 2 and 3 were identified. Unbalanced process 1 described the enriched pathways following the response of the cells after IR at 2 Gy for 48 h. These pathways included the PI3K/mTOR, AMPK and autophagy pathways (Fig. 3A and Supplementary Fig. S3A). Unbalanced process 1 indicated the coexpression and induction of different proteins, including p38-MAPK, ASNS, FGF, and GATA6, in response to irradiation at a dose of 2 Gy for 48 h. As mentioned in the material and methods, the results are depicted as an amplitude (negative/positive amplitude indicates the correlation of cells with respect to a specific process—Figure 3A–left), STRING analyses (Fig. 3A–right), and a network of proteins (Supplementary Fig. 3A). Unbalanced process 2 described the responses of the cells after 72 h of KDM5B overexpression (+ dox) (Fig. 3B, Supplementary Fig. S3B). In KDM5B-overexpressing cells, the network with induced pAKT/pS6/VEGFR2 (among other proteins labeled in red in the network) was dominant at this time point (Supplementary Fig. 3B-right). Unbalanced process 3 described the enriched pathways following the response of the cells after hypoxia (Hx 10) at 24 h and 48 h. (Fig. 3C, Supplementary Fig. S3C). This process highlighted the enrichment of genes related to DNA damage responses and the TGF-beta and cellular senescence pathways. Our findings corroborate the presence of common resistance mechanisms and establish the role of KDM5B in the development of acquired IR resistance. Furthermore, KDM5B overexpression is dependent on distinct signaling pathways (e.g., the AKT/PI3K pathway), highlighting potential therapeutic vulnerabilities that can be strategically targeted. PI3K inhibition in combination with IR resulted in increased killing efficiency of cycling hypoxia-adapted cells. To confirm the results of RPPA-based PaSSS, which revealed KDM5B-dependent activation of the AKT signaling pathway in hypoxia-adapted and irradiated cells, we treated WM3734^KDM5B^ and WM3734^n^ melanoma cells with one of the following two sequential combination treatments. We either treated the cells with the PI3K inhibitor GSK458 or the AKT inhibitor MK2206 (referred to as TT-targeted therapy) followed by IR (0, 2, or 5 Gy), or vice versa (Fig. 4A). Each treatment was given for 3 days; for example, the cells were treated with TT for 3 days and then further irradiated with a single dose on day 3 (0, 2, or 5 Gy). The readouts were performed 3 days after the second treatment (Fig. 4A). For the repopulation assay, cells were counted on day 6 and then seeded at equal densities, and the medium was changed once/week without treatment. The ability of the cells to repopulate was subsequently monitored. The assay was stopped when at least one well was ~ 100% confluent in the IR→TT sequence + dox (this sequence has a faster growth rate than the TT→IR sequence). To compare the different treatment conditions without bias [1], the sequence IR→TT and sequence TT→IR of one cell line and one inhibitor were treated as one experiment and stopped when one well reached ~ 100% confluence [2]. We seeded WM3734^KDM5B^*in the presence of dox* and then either removed it when performing the repopulation assay or kept it (Fig. 4A). To assess the cell density in each well, we introduced an intensity score, where a score of 4 indicates 70–90% confluence, whereas a score of 0 indicates ≤ 9% confluence. A representative example is shown in Fig. 4B.

The repopulation assay revealed differences between the two treatment sequences. The sequence TT→IR (GSK458 or MK2206) was more efficient at eliminating melanoma cells than the IR→TT sequence was (Fig. 4D, E compared with F, G). This was apparent when the combination therapy wells in both sequences were compared. Importantly, when the intensity scores were compared, the combination of GSK458 and 5 Gy (but not MK2206 or 5 Gy) affected the regrowth of Hx 10 WM3734^KDM5B^ cells and was more efficient at reducing the repopulation ability of Hx 10 WM3734^KDM5B^ cells than Ctl (black box) at a concentration of [0.1 µM] (Fig. 4B). In detail, Fig. 4B (black box) is depicted in Fig. 4C as a heatmap. A reduction in repopulation ability after treatment was demonstrated by a reduction in the intensity score from 1 to 0 in Ctl and 3 to 1 in Hx 10 cells. However, the repopulation ability of the cells treated with MK2206 was less affected (Fig. 4F, G). These findings indicate that, compared with the Ctl cells, the PI3K pathway might be more essential for supporting Hx10 repopulation ability (at the concentrations used).

Next, to assess the importance of KDM5B for cell survival following the different sequences, we removed dox upon seeding for the repopulation assay (Fig. 4B, lower panel). We noticed that the removal of dox (removal of KDM5B overexpression) in WM3734^KDM5B^ Hx 10 cells resulted in a strong decrease in repopulation ability in the GSK458 sequences but not in the MK2206 sequences (blue boxes (Ctl dox/removal of dox) compared with red boxes (Hx 10 dox/removal of dox)), Fig. 4D, E, G. No decrease in repopulation ability was observed following dox removal, as shown in Fig. 4F.

Next, we performed similar experiments using WM3734^n^ cell lines (Supplementary Fig. S4). We observed that a similar concentration of GSK458 was more efficient than MK2206 at affecting the repopulation abilities of melanoma cells regardless of the treatment sequence (Supplementary Fig. S4A, C compared with B, D). We did not observe a significant repopulation advantage for WM3734^n^ Hx 10 cells compared with the control.

To confirm the dependence on the PI3K pathway, we knocked down p110α using siRNA for 72 h in WM3734^KDM5B^ Ctl and Hx 10 cell lines, then we irradiated the cells with 0 and 2 Gy (with and without dox). After another 72 h, we assessed [i] the efficiency of the KD and [ii] cell death using trypan blue exclusion staining. In Supplementary Fig. S4E, p110α was KD as shown on the protein level. In Fig. 4 (H, I), at 0 Gy, we saw a significant increase in the percentage of dead cells in Hx 10 following p110 α KD and under dox addition (overexpression of KDM5B), compared to Ctl cells (Fig. 4H). Similar observations were evident when the ratio of dead to living cells was compared, where Hx 10 cell showed a higher dead to live cell ratio when p110α was knocked down (Fig. 4I). This is likely due to the dependence of Hx 10 cells on the PI3K pathway, especially under KDM5B OE.

**Fig. 5:**
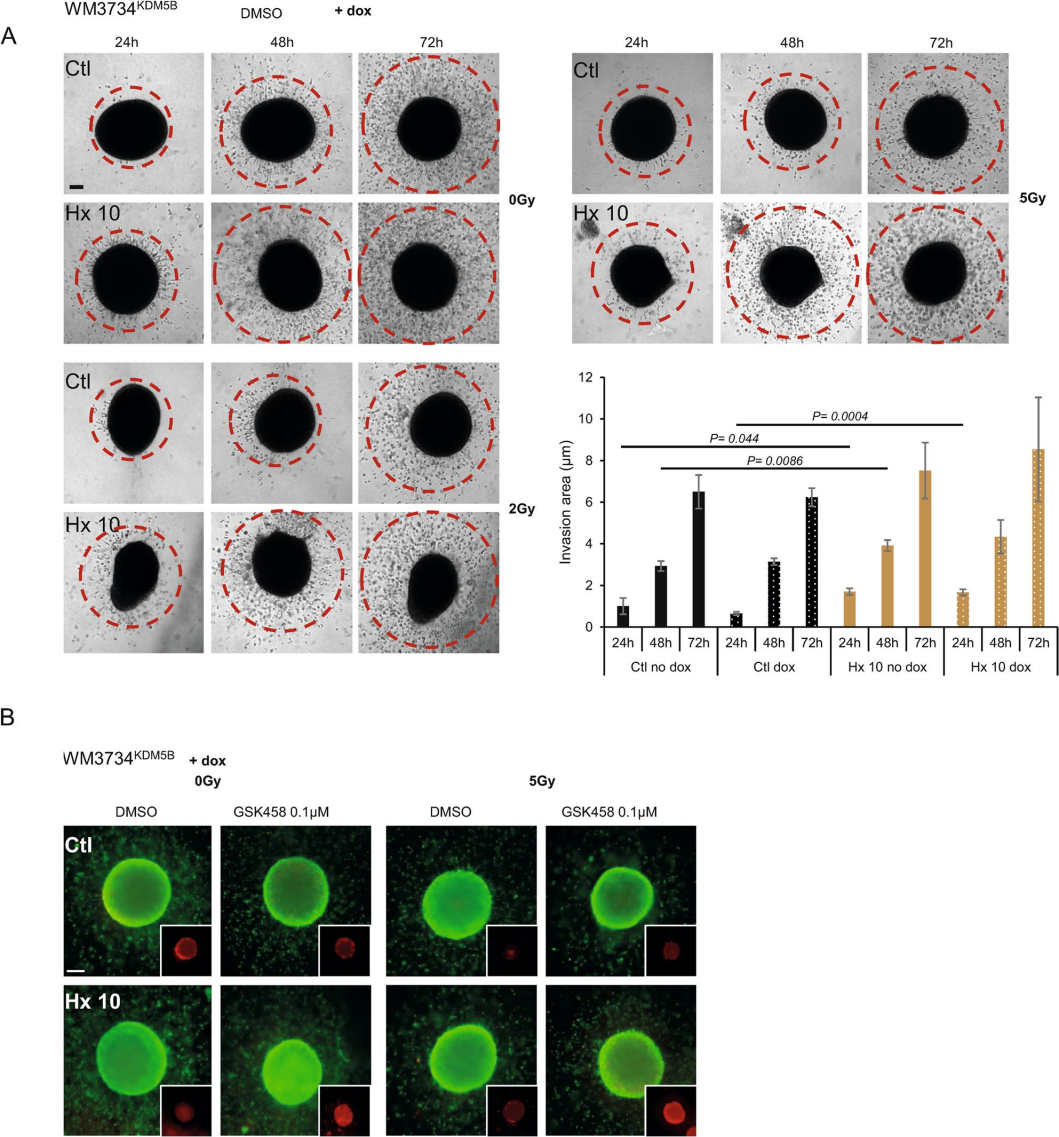
Different sequential treatments affect the repopulation of melanoma cell lines. **A** 3D collagen embedded spheroids of WM3734^KDM5B^ Ctl and Hx 10 in the presence of dox (KDM5B overexpression). Phase images are shown following DMSO treatment and different IR doses (0, 2, 5 Gy) over 24, 48, and 72 h. Red circles indicate the extent of single cell migration from the spheroid. Scale bar 200 μm (**B**) Live/dead staining (live: green, dead: red) after 72 h of GSK458 [0.1 µM] and different IR doses (0, 5 Gy). Inserts indicate red signal intensities. The experiments were repeated n = 3 times unless otherwise mentioned

To better mimic the tumor microenvironment, we validated our findings in 3D collagen-embedded spheroids. We grew our cells as 3D collagen-embedded spheroids, treated them with GSK458 [0.1 µM], and then irradiated them- and imaged over 3 days. On day 3 after treatment, we added live/dead staining. In Fig. 5A, phase images of DMSO- and IR-treated WM3734^KDM5B^ Ctl and Hx 10 cell lines *in the presence of dox* (KDM5B overexpression) over time showed more invasion (individual cells migrating from the spheroid- red circles) in Hx 10 cells compared to Ctl. Live/dead staining (green: live, red: dead) 72 h after treatment (GSK458 and RT) was performed in Fig. 5B. Red inserts depicted the dead cell signal. Interestingly, our 3D data validated our 2D data, where Hx 10 cells demonstrated more cell death following GSK458 treatment compared to Ctl and DMSO (especially in 0 Gy and 5 Gy following GSK458), quantification is shown in Fig. 5A (invasion area was significantly increased in Hx 10 compared to Ctl after 24 h and 48 h without dox addition and after 24 h under the presence of dox). Supplementary Fig. 5 depicted the naïve cells WM3734^n^, and as confirmed with 2D models, 3D collagen-embedded spheroids showed slightly more invasion in Hx 10 compared to Ctl cells (Supplementary Fig. S5A), while little to no changes were seen following the live/dead staining (Supplementary Fig. S5B).

Our results provide valuable insights into how (i) different sequential combination therapies and (ii) expression levels of KDM5B affect the survival and repopulation of melanoma cell subpopulations that have adapted to cycling hypoxia and thereby gained cross-resistance to radiation therapy and raise the question of how this might translate to patients in the clinic. Extensive validation is still needed; however, this work highlights the importance of using different sequences to assess therapeutic efficacy.

**Fig. 6:**
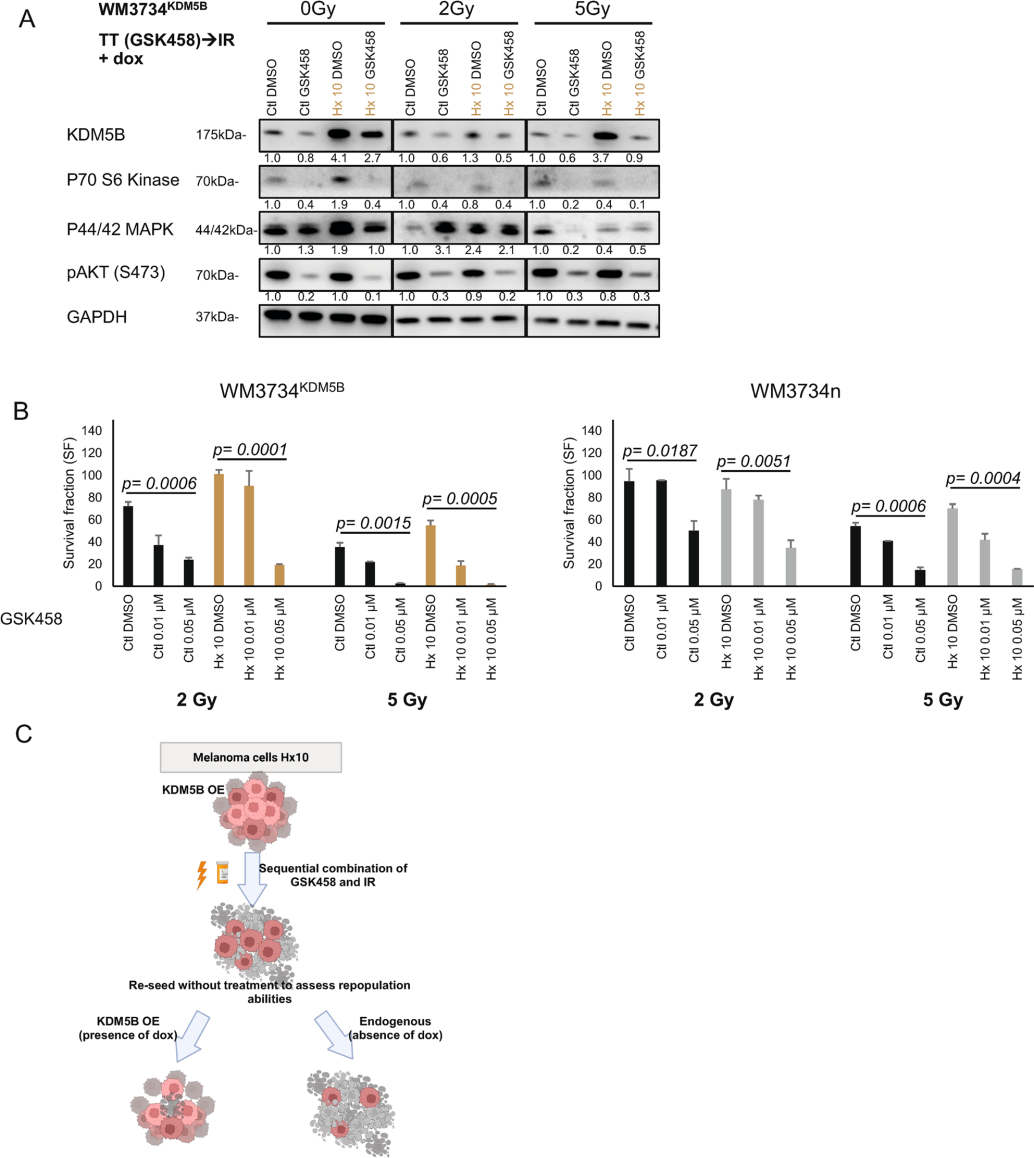
KDM5B protects against the effects of IR. **A** WB of WM3734^KDM5B^ melanoma cells with dox after TT (GSK458)→IR. **B** Limiting dilution assay. The number of colonies formed after seeding 1,000, 500, 250, 125, 62.5, 31.25, 15.6, 7.8 WM3734^KDM5B^ and WM3734^n^ melanoma cells/well into 96-well plates is shown. The cells were treated with GSK458 (0.01 or 0.05 µM) for 24 h and then irradiated with a single dose (0, 2, or 5 Gy) for 24 h. The cells were cultured for 10‒30 days, fixed, stained (containing blue) and counted. The software assumed that 0 Gy of DMSO would result in 100% cell survival. The survival fraction was then calculated. Ordinary one-way ANOVA was performed for the statistical analyses. The experiments were repeated *n* = 3 times unless otherwise mentioned. The bar graphs present the means ± SDs. **C** Overexpression of KDM5B in resistant Hx 10 melanoma cell lines increased survival and repopulation ability. Combination sequential treatment decreased the survival of these cells, and the removal of overexpressed KDM5B further decreased their repopulation ability

Since the PI3Ki GSK458 was more efficient at decreasing the repopulation ability of cells than the AKTi MK2206 was, we performed a western blot analysis of WM3734^KDM5B^ melanoma cells *in the presence of dox* to confirm that downstream targets of the PI3K pathway were inhibited (Fig. 6A). Western blot analysis revealed the inhibition of p70 S6K, p44/42 MAPK and pAKT (S473) following GSK458 treatment (Fig. 6A). However, IR treatment (second treatment) further inhibited the downstream signaling pathways in all the cell lines (Fig. 6A). These data highlight the importance of sequential combination therapies in inhibiting cancer signaling pathways.

Finally, we performed a limiting dilution assay (LDA) to assess the long-term ability of both cell lines, WM3734^KDM5B^ and WM3734^n^, to form colonies. We treated the cells with either 0.01 µM or 0.05 µM GSK458 and then irradiated them with 0, 2, or 5 Gy in the absence of dox. The treatment mixture was added only once the cells reached ~ 70% confluence. The survival fraction was calculated via normalization to 0 Gy of DMSO (100% survival). All the treatments resulted in significant survival differences between the DMSO-treated group and the 0.05 µM GSK458-treated group. However, the data indicated that at 5 Gy in combination with 0.05 µM GSK458 treatment, WM3734^KDM5B^ Hx 10 cells were the least surviving cell line, confirming the ability of the sequential combination treatment to overcome the adaptive/resistant phenotype observed in WM3734^KDM5B^ Hx 10 cells (Fig. 6B).

These results confirm [i] the importance of KDM5B in mediating adaptation to intermittent cycling hypoxia and subsequent cross-resistance to radiation therapy and [ii] the importance of sequential combination therapy together with KDM5B inhibition as a therapeutic strategy to target therapy-resistant subpopulations.

## Discussion

Melanoma is known for its high degree of tumor heterogeneity (mainly caused by genetic and epigenetic mechanisms) and ability to undergo phenotype switching (dynamic cell state transitions that involve reversible transcriptional changes and epigenetic modifications) [47, 48].

Clinically, hypoxia is a key driver of melanoma progression, therapeutic resistance, and immune evasion. By targeting hypoxia-induced pathways (e.g., PI3K) and the associated tumor microenvironment changes, there is significant potential to improve melanoma treatment outcomes [16]. Additionally, significant efforts have been made to develop therapies to target tumor hypoxia, e.g., VEGF and HIF1 inhibitors, which have been met with limited success rates [49, 50]. Despite these efforts, a complete understanding of intermittent cycling hypoxia biology and how it affects resistance in melanoma is still lacking. Using an information-theoretic, SA-based computational approach to analyze RPPA data, we characterize individualized network alterations to identify synthetic vulnerabilities related to KDM5B OE, adaptation to cycling hypoxia or both, in melanoma following radiation.

PaSSS (protein-specific signaling signature) combined with STRING pathway enrichment analyses revealed distinct sets of dysregulated signaling pathways in WM3734^KDM5B^ (KDM5B OE) Hx 10 HRT cells compared with their nonadapted Ctl cells, which were specifically related to intermittent cycling hypoxia and KDM5B-driven signaling alterations (e.g., PI3K). These distinct sets of dysregulated signaling pathways are called processes. These processes are unique and lead us to hypothesize that KDM5B overexpression facilitates adaptation to intermittent cycling hypoxia conditions, thereby activating signaling pathways that confer resistance to irradiation through activation of the DNA damage response, MAPK, and PI3K/mTOR pathways.

Our data demonstrated that following intermittent cycling hypoxia, there was an increase in the baseline expression of KDM5B in WM3734^KDM5B^ Hx 10 HRT cells compared with that in nonselected control (Ctl) cells. Our previous work and the work of others revealed that KDM5B is a slow cell cycle marker and has a role in melanoma resistance to various therapies, including RT [24, 26]. Our current work demonstrated the importance of KDM5B in mediating radiation tolerance and resistance. KDM5B overexpression was a major contributor to altered signaling pathways in unbalanced process 2. Moreover, we demonstrated that WM3734^KDM5B^ Hx 10 HRT cells harbor distinct PaSSSs (unbalanced process 2) with increased DNA repair capabilities and activation of the PI3K, HIF-1, and AMPK signaling pathways. Moreover, unbalanced process 1 was driven by an enrichment in the PI3K/mTOR pathway following IR treatment for 48 h. This finding confirms that while some of the identified enriched pathways are commonly associated with resistance processes, others are specific to the radiation response, intermittent cycling hypoxia or KDM5B overexpression. The manipulation of KDM5B (OE) has revealed its importance in cycling hypoxia tolerance development and subsequent cross-resistance to IR. Furthermore, a study by Bayo et al. demonstrated that, in lung cancer, the catalytic activity of KDM5B is essential for the efficient and complete repair of double-strand breaks following radiation, and the inhibition of KDM5B activity impaired the recruitment of key repair factors [28].

Our cell models revealed that there are differences in how the cells respond to the sequential combination treatments. The WM3734^KDM5B^ KDM5B OE cells demonstrated a dependence on KDM5B for survival after sequential combination treatment, which was proven by the removal of doxycycline as an inducer of KDM5B OE; the cells had markedly reduced abilities to repopulate upon sequential combination therapies, including IR. In comparison, the naïve WM3734^n^ cells did not demonstrate such variations or vulnerabilities because they expressed only endogenous KDM5B.

The results presented here reveal the utility of an RPPA screen combined with PaSSS analysis in identifying synthetic lethal interactions, providing a proof-of-concept for potential applications in melanoma therapy. Specifically, KDM5B is upregulated in cells resistant to intermittent cycling hypoxia (Hx 10), driving the enrichment of several established and novel survival pathways in melanoma. Importantly, the radiosensitization observed here may reflect a form of synthetic vulnerability, where inhibition or loss of KDM5B compromises homologous recombination (via reduced BRCA1/2, RAD51, and RPA1 activity) while also diminishing prosurvival PI3K signaling.

GSK458 (also known as GDC-0941) is a class I PI3K inhibitor with known activity upstream of AKT and broader influence on PIP3-dependent signaling [51]. MK2206 is an allosteric AKT inhibitor acting downstream. Several studies show that PI3K inhibition produces a more complete suppression of AKT signaling and parallel pathways such as mTORC1/2 or SGK1, whereas AKT-specific inhibitors allow compensatory feedback loops [52]. Based on our data, the stronger effect of GSK458 likely reflects its upstream position within the PI3K/AKT pathway. As a PI3K inhibitor, GSK458 suppresses multiple downstream signalling nodes simultaneously, whereas MK2206 acts further downstream and cannot fully block PI3K-dependent compensatory signalling. These data were supported in our 3D models, demonstrating more killing efficiency in Hx 10 cells compared to Ctl following KDM5B overexpression. This interpretation is supported by our Western blot data, which show broader inhibition of key PI3K-regulated effectors (including AKT, p70S6K and p44/42 MAPK) after GSK458 treatment. Functionally, this broader pathway engagement mirrors the more pronounced effects observed in our repopulation assays across both melanoma models and treatment sequences. In early clinical trials of GDC-0941 (pictilisib), a patient with V600E BRAF-mutant melanoma experienced a confirmed partial response, indicating that PI3K inhibition can show activity in melanoma under certain conditions [53].

As a consequence, melanoma cells exposed to radiotherapy may accumulate unrepaired DNA lesions, undergo mitotic catastrophe, or undergo apoptosis. This is particularly relevant in the context of BRAFV600-mutant melanoma, where resistance to targeted therapies is often mediated by adaptive upregulation of stress pathways. KDM5B inhibition could therefore synergize with radiotherapy by simultaneously blocking adaptive transcriptional programs and enhancing genomic instability. In line with the role of KDM5B in DNA damage processing, several of our findings point toward altered genomic maintenance in hypoxia-adapted melanoma cells. Although we did not perform dedicated genomic instability assays such as micronucleus formation or karyotyping, our analyses already reveal multiple surrogate markers consistent with impaired DNA repair dynamics. Hx 10 cells exhibit reduced clearance of γH2AX foci upon KDM5B withdrawal, and our RPPA profiling demonstrates pronounced alterations in key DNA repair-associated proteins, including RAD51, PARP2, FEN1, H2AX and KDM5C. Functionally, these molecular signatures align with the diminished survival and repopulation capacity observed after sequential PI3K inhibition and irradiation, suggesting a heightened reliance on intact DNA repair pathways for stress tolerance. Together, these results support the hypothesis that hypoxia-adapted and KDM5B-high melanoma cells harbor a therapeutically exploitable vulnerability in genomic stability. Future studies integrating direct genomic instability measurements will further refine this mechanistic understanding and may help identify biomarkers predictive of therapeutic response.

Our work emphasizes the importance of developing approaches to assess sequential combination therapies and different treatment schedules in melanoma patients and demonstrates how different treatments can lead to different survival outcomes in melanoma patients. Our data demonstrated that KDM5B overexpression in resistant Hx 10 melanoma cell lines allows them to have a survival advantage and increased repopulation ability. The combination of PI3Ki (GSK458) and IR decreases the survival of these cells, and the removal of overexpressed KDM5B further reduces their repopulation ability (Fig. 5C). Further studies are needed to [i] confirm the role of KDM5B as a therapeutic vulnerability factor in melanoma cells adapted to cycling hypoxia and resistant to RT and [ii] identify the key players within the signaling pathways highlighted. One limitation of our work is that it was performed in 2D cell culture.

Lastly, although our study focuses on melanoma-specific vulnerabilities, we recognize that the potential toxicity of combined KDM5B and PI3K inhibition in normal melanocytes and other non-malignant cells requires careful evaluation. Normal melanocytes typically express low levels of KDM5B and do not acquire the persister-like, therapy-tolerant states observed in melanoma, and PI3K/AKT signalling is less strongly activated in non-malignant cells, suggesting that a therapeutic window may exist. Nonetheless, dedicated toxicity studies will be essential to determine the safety and translational feasibility of this combinatorial approach, and we highlight this as an important direction for future work. The next steps will involve confirming our hypothesis in 3D and in vivo models. Future work should evaluate whether KDM5B inhibition selectively sensitizes tumor cells while sparing normal melanocytes, as well as whether combining epigenetic therapy with radiotherapy produces durable responses in vivo. Integrating our proteomic data with transcriptional and chromatin accessibility profiling would also help clarify how KDM5B orchestrates the balance between DNA repair and cell survival under genotoxic stress.

## Conclusions

This study identifies KDM5B as a critical mediator of melanoma cell adaptation to intermittent cycling hypoxia and a contributor to radiation resistance. By integrating proteomic profiling with information-theoretic analysis, we uncovered distinct signaling signatures that highlight how KDM5B-driven proteomic changes in DNA repair and survival pathways promote tolerance to radiotherapy. Importantly, our work demonstrates that inhibition of KDM5B and the PI3K pathway can reduce this resistance, suggesting that combined targeting strategies may expose synthetic vulnerabilities in hypoxia-adapted melanoma cells. These findings provide new insight into the interplay between epigenetic regulation, hypoxia-induced signaling, and therapy response, with potential implications for the development of more effective and durable treatment strategies in melanoma. Future validation will include the use of stable KD of p110α to study the mechanism of action between KDM5B and PI3K in melanoma, as well as more mechanistic insights through proteomic screens such as ChIPseq.

For future clinical translation, stratifying patients who may benefit from KDM5B-targeted approaches will be essential. KDM5B can be reliably assessed by immunohistochemistry and has been consistently associated with slow-cycling, therapy-tolerant melanoma cell states, making its expression a promising starting point for patient selection. Because hypoxia strongly shapes these KDM5B-dependent phenotypes, incorporating hypoxia-associated biomarkers, such as CAIX or HIF-1α, or functional hypoxia imaging, may further refine stratification. Although dedicated biomarker development was beyond the scope of this study, combining KDM5B expression with indicators of tumor hypoxia may ultimately help identify patients most likely to benefit from such interventions, and we highlight this as an important direction for future translational work.

## Supplementary Information

Supplementary Material 1: Figure S1. (A) Cell cycle/PI analyses over a time course after 0, 2, or 5 Gy of irradiation of the cell lines in Fig. 1A. The bar graphs present the means ± SDs from n = 2 independent experiments. (B) Cell cycle/PI analyses over a time course after 0, 2, or 5 Gy following KDM5B inhibition (siKDM5B). The bar graphs present the means ± SDs from n = 2 independent experiments. (C) Western blot analyses showing KDM5B expression following radiation over a time course. The same color boxes indicate points of comparison. The numbers indicate the signal intensity normalized to that of DMSO and the loading marker.

Supplementary Material 2: Figure S2. (A) Heatmap showing protein expression levels (log2 normalized) of proteins that overlap with Fig. 2A in WM3734^n^ Hx 10 cell lines following the targeted RPPA. (B) Volcano plots visualizing the differences in protein expression (log2-fold change) and q values (-log10 adjusted p values) in WM3437^n^. (C) Gamma H2AX foci staining following IR in WM3734^n^ melanoma cell lines. (D) Quantification of Gamma H2AX foci following IR in WM3734^n^ melanoma cell lines. The experiments were repeated n = 3 times unless otherwise mentioned.

Supplementary Material 3: Figure S3. STS of the up- and downregulated proteins in unbalanced processes 1 (A), 2 (C), and 3 (C). The network maps are on the right-hand side.

Supplementary Material 4: Figure S4. (A -D) Repopulation assay following sequential combination treatment (TT→ IR and IR→TT) in WM3734^n^ melanoma cell lines. GSK458 and MK2206 were used at 0.01 µM. The cells were treated with sequential combination therapy (TT→ IR and IR→TT). GSK458 or MK2206 (0.1 µM) was given for 3 days, after which the cells were treated with 0–5 Gy of IR for another 3 days (and vice versa), after which a repopulation assay was performed. The repopulation assay was stopped when at least one well reached ~ 100% confluence. The experiments were repeated n = 3 times unless otherwise mentioned. The bar graphs present the means ± SDs. (E) WB depicting the knockdown of p110α using 20nM siRNA in WM3734KDM5B melanoma cell lines.

Supplementary Material 5: Figure S5. (A) 3D collagen embedded spheroids of WM3734^n^ Ctl and Hx 10 in the presence of dox (KDM5B overexpression). Phase images are shown following DMSO treatment and different IR doses (0, 2, 5 Gy) over 24, 48, and 72 h. Red circles indicate the extent of single cell migration from the spheroid. Scale bar 200 μm (B) Live/dead staining (live: green, dead: red) after 72 h of GSK458 [0.1 µM] and different IR doses (0, 5 Gy. Inserts indicate red signal intensities. The experiments were repeated n = 3 times unless otherwise mentioned. Significance was tested via Student’s t test.

Supplementary Material 6.

Supplementary Material 7. Uncut WB membranes.

## Data Availability

Requests for further information and resources should be directed to and will be fulfilled by the lead contact Batool Shannan (Batool.shannan@uk-essen.de).
