# Supplementary material for: The role of KDM5B in creating synthetic vulnerabilities in combination with radiotherapy in melanoma cells
Source: Cell Commun Signal. 2026 Mar 5;24:212. doi: 10.1186/s12964-026-02714-5 (PMC13064131; doi:10.1186/s12964-026-02714-5)
Supplement: Supplementary file 6 — Supplementary Material 6. [file 12964_2026_2714_MOESM6_ESM.docx]

**The role of KDM5B in creating synthetic vulnerabilities in combination with radiotherapy in melanoma cells**

Safa Larafa^1^, Merle Schaffrin^1^, Peer Braß^2^, Renáta Váraljai^3,4^, Sarah Scharfenberg^3,4^, Nataly Kravchenko-Balasha^5^, Gil Polinovski^5^, Meenhard Herlyn^6^, Nooraldeen Tarade^7^, Stefan Wiemann^7^, Alexander Roesch^3,4^, Dirk Schadendorf^3,4^, Verena Jendrossek^1, 4, 7^, Johann Matschke^1, 4^*, Batool Shannan^3,4^*

^1^Institute of Cell Biology (Cancer Research), University of Duisburg-Essen, Medical School, Germany. ^2^Department of Infectious Diseases, West German Centre of Infectious Diseases, University Duisburg-Essen, 45147 Essen, Germany. ^3^Department of Dermatology, University Hospital Essen, West German Cancer Center, University Duisburg-Essen, Essen, Germany. ^4^German Consortium for Translational Cancer Research (DKTK), Partner Site Essen/Düsseldorf, Essen/Düsseldorf, Germany. ^5^The Institute of Biomedical and Oral Research, Hebrew University of Jerusalem, Jerusalem 91120, Israel. ^6^The Wistar Institute, Philadelphia, PA, 19104, USA. ^7^ Division of Molecular Genome Analysis, German Cancer Research Center (DKFZ), Heidelberg, Germany. ^7^ West German Comprehensive Cancer Center Essen (CCC-WTZ).


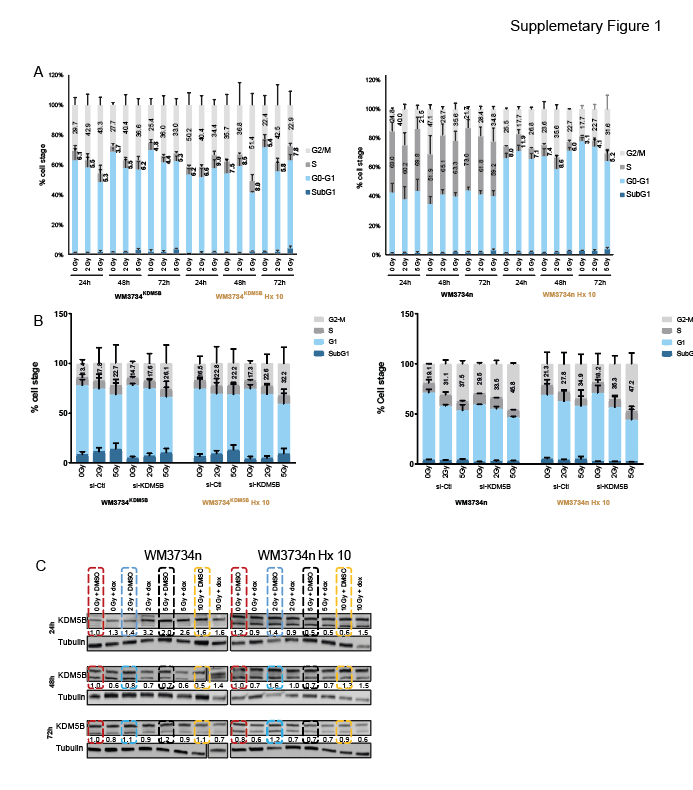


***Supplementary Figure 1. Related to Figure 1****. (A) Cell cycle/PI analyses over a time course after 0, 2, or 5 Gy of irradiation of the cell lines in Figure 1A. The bar graphs present the means ± SDs from n=2 independent experiments. (B) Cell cycle/PI analyses over a time course after 0, 2, or 5 Gy following KDM5B inhibition (siKDM5B). The bar graphs present the means ± SDs from n=2 independent experiments. (C) Western blot analyses showing KDM5B expression following radiation over a time course. The same color boxes indicate points of comparison. The numbers indicate the signal intensity normalized to that of DMSO and the loading marker.*


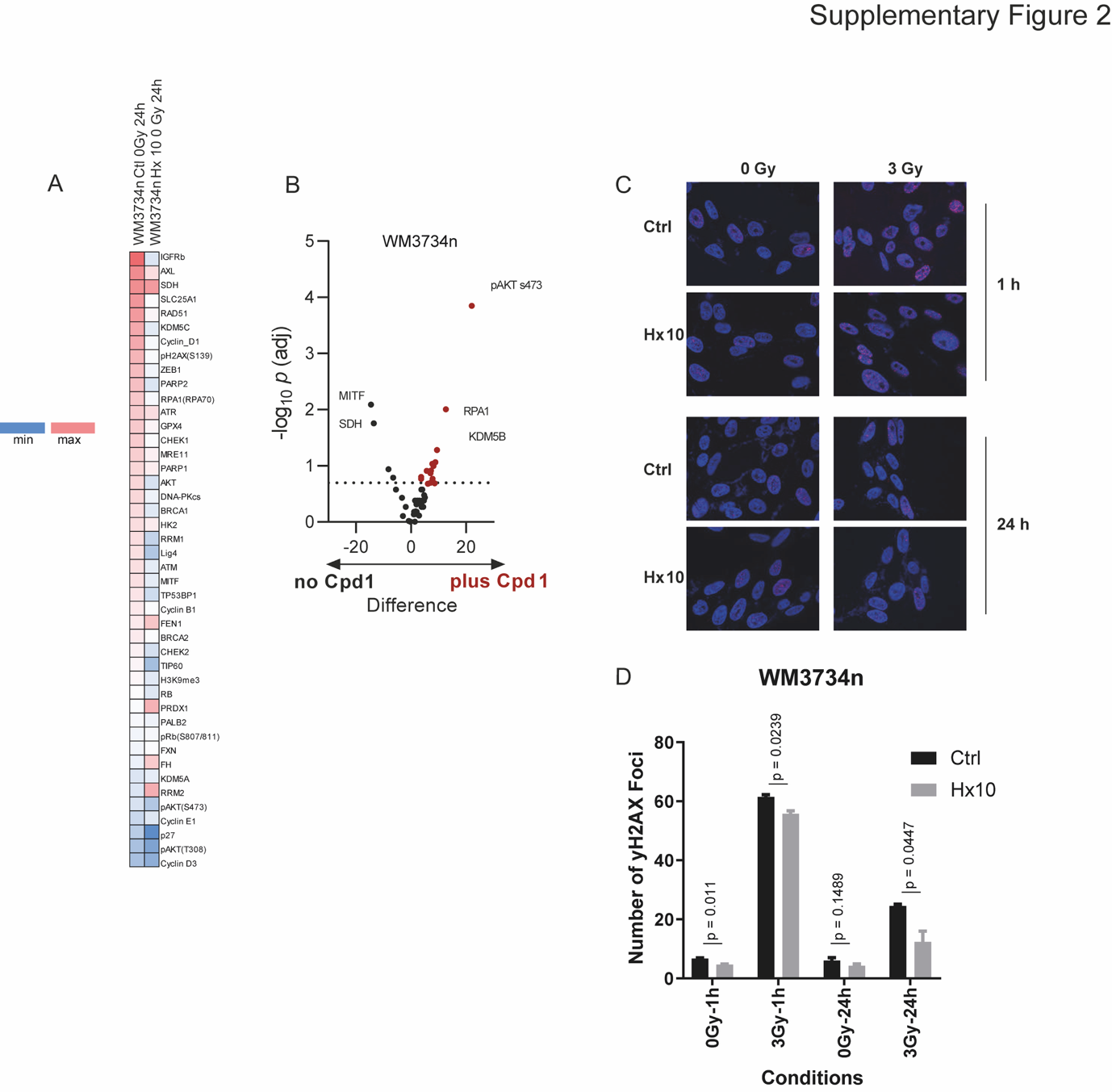


***Supplementary Figure 2. Related to Figure 2****. (A) Heatmap showing protein expression levels (log_2_ normalized) of proteins that overlap with Figure 2A in WM3734n Hx 10 cell lines following the targeted RPPA. (B) Volcano plots visualizing the differences in protein expression (log_2_-fold change) and q values (-log_10_ adjusted p values) in WM3437n. (C) Gamma H2AX foci staining following IR in WM3734n melanoma cell lines. (D) Quantification of Gamma H_2_AX foci following IR in WM3734n melanoma cell lines. The experiments were repeated n=3 times unless otherwise mentioned.*


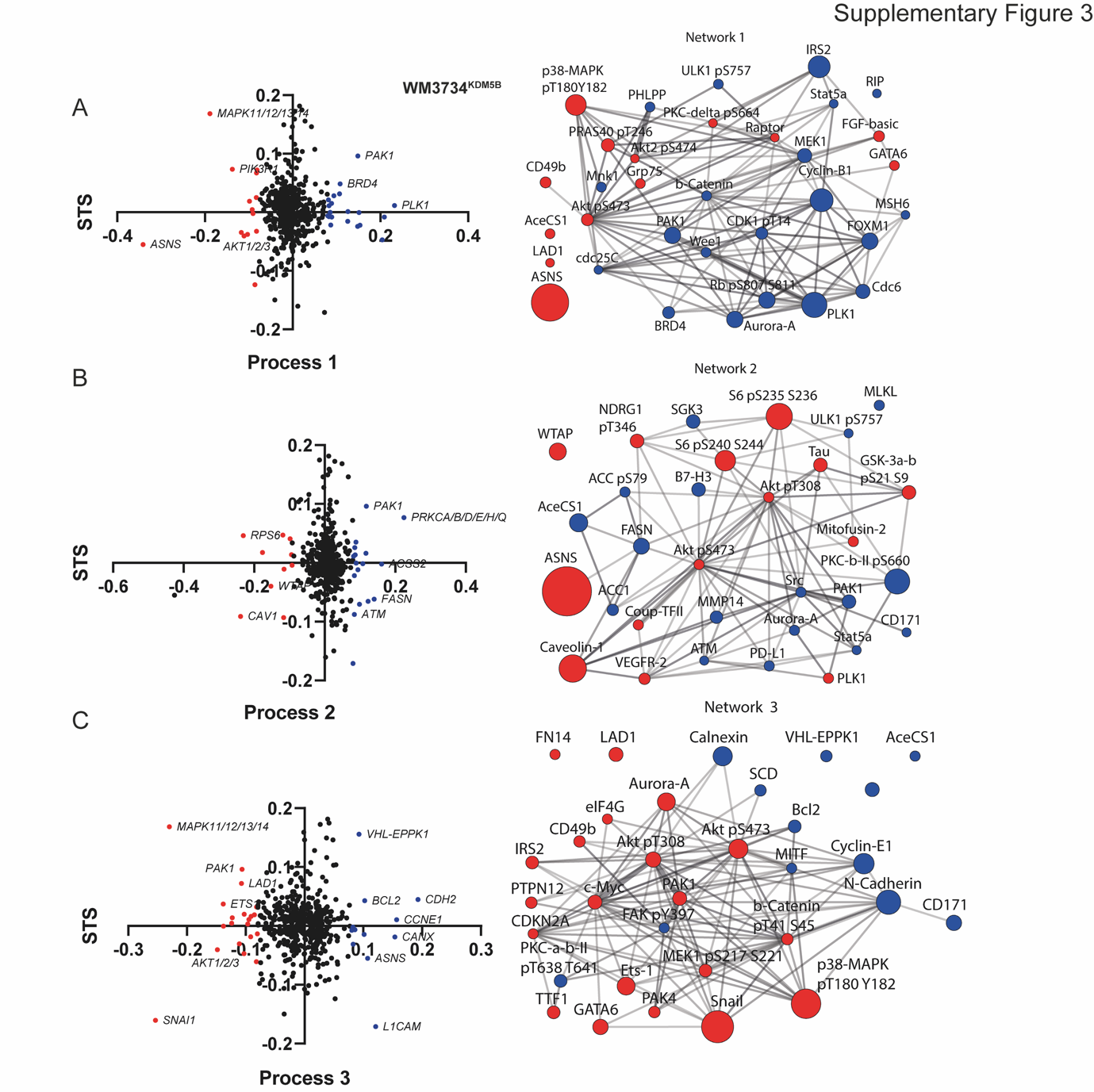


***Supplementary Figure 3. Related to Figure 3.*** *STS of the up- and downregulated proteins in unbalanced processes 1 (A), 2 (C), and 3 (C). The network maps are on the right-hand side.*


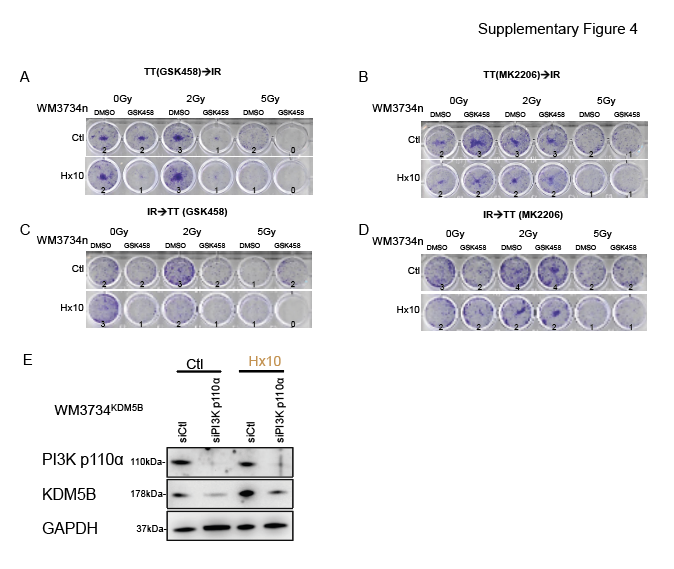


***Supplementary Figure 4. Related to Figure 4.*** *(A -D) Repopulation assay following sequential combination treatment (TT🡪 IR and IR🡪TT) in WM3734n melanoma cell lines. GSK458 and MK2206 were used at 0.01 µM. The cells were treated with sequential combination therapy (TT🡪 IR and IR🡪TT). GSK458 or MK2206 (0.1 µM) was given for 3 days, after which the cells were treated with 0 or 5 Gy of IR for another 3 days (and vice versa), after which a repopulation assay was performed. The repopulation assay was stopped when at least one well reached ~100% confluence. The experiments were repeated n=3 times unless otherwise mentioned. The bar graphs present the means ± SDs.* *(E) WB depicting the knockdown of p110α using 20nM siRNA in WM3734KDM5B melanoma cell lines.*

*
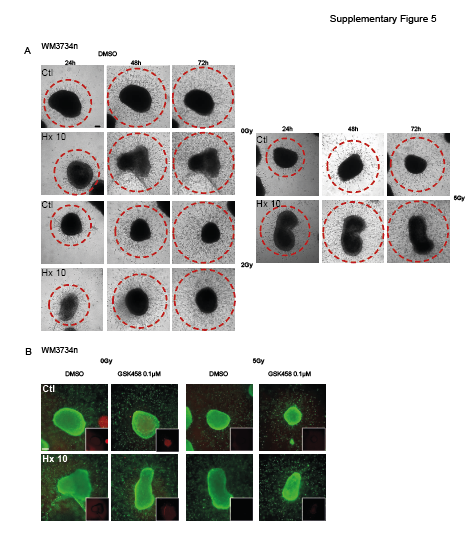
*

***Supplementary Figure 5. Related to Figure 5.*** *(A) 3D collagen embedded spheroids of WM3734n Ctl and Hx 10 in the presence of dox (KDM5B overexpression). Phase images are shown following DMSO treatment and different IR doses (0, 2, 5 Gy) over 24, 48, and 72h. Red circles indicate the extent of single cell migration from the spheroid. Scale bar 200 µm (B) Live/dead staining (live: green, dead: red) after 72h of GSK458 [0.1 µM] and different IR doses (0, 5 Gy. Inserts indicate red signal intensities. The experiments were repeated n=3 times unless otherwise mentioned. Significance was tested via Student’s t test.*
