# Supplementary material for: The role of KDM5B in creating synthetic vulnerabilities in combination with radiotherapy in melanoma cells
Source: Cell Commun Signal. 2026 Mar 5;24:212. doi: 10.1186/s12964-026-02714-5 (PMC13064131; doi:10.1186/s12964-026-02714-5)
Supplement: Supplementary file 7 — Supplementary Material 7. Uncut WB membranes. [file 12964_2026_2714_MOESM7_ESM.pptx]

## Slide 1
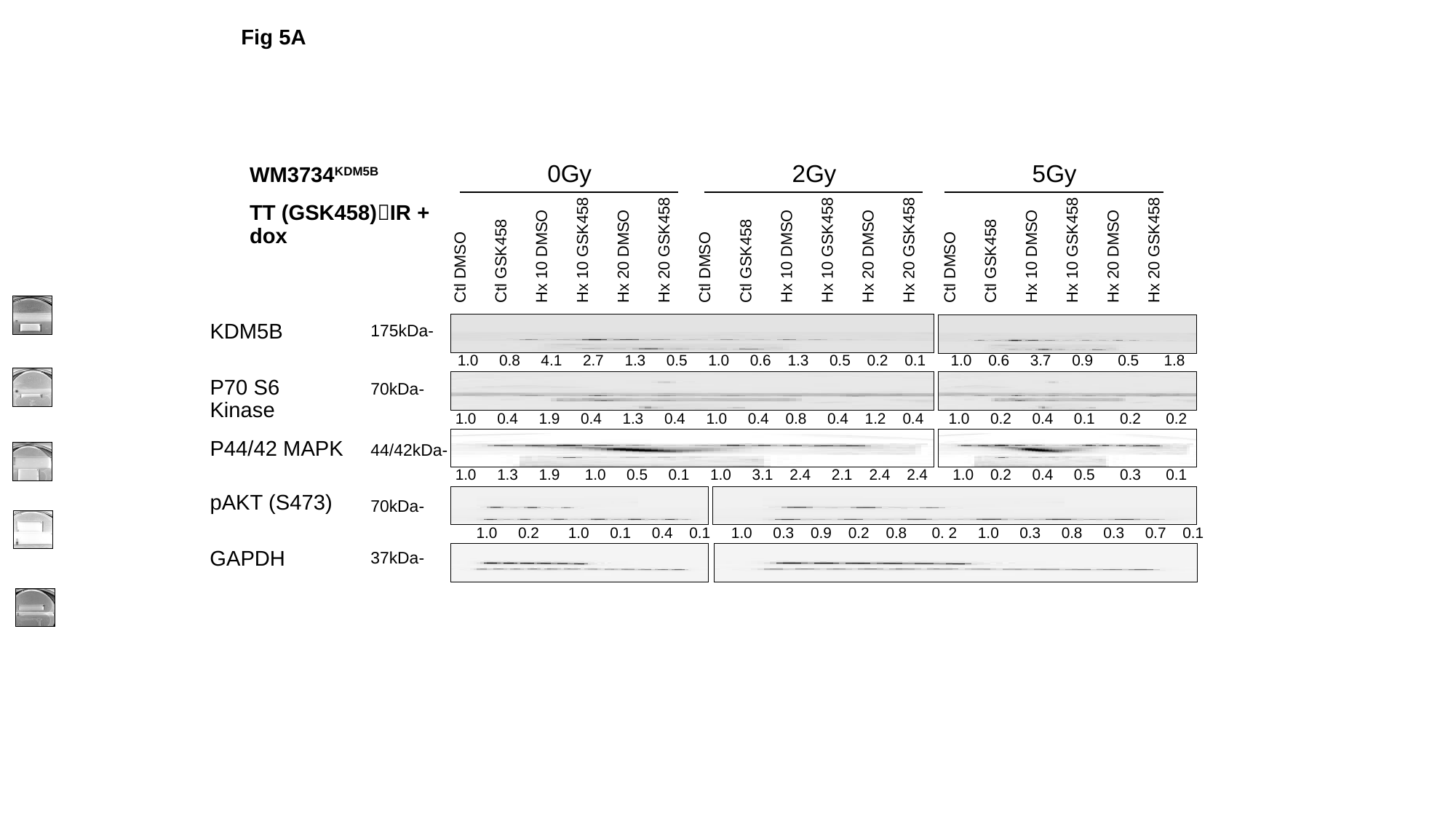

Fig 5A
0Gy
2Gy
5Gy
WM3734KDM5B
TT (GSK458)IR + dox
Hx 10 GSK458
Hx 20 GSK458
Hx 10 GSK458
Hx 20 GSK458
Hx 10 GSK458
Hx 20 GSK458
Hx 10 DMSO
Hx 20 DMSO
Hx 10 DMSO
Hx 20 DMSO
Hx 10 DMSO
Hx 20 DMSO
Ctl GSK458
Ctl GSK458
Ctl GSK458
Ctl DMSO
Ctl DMSO
Ctl DMSO
KDM5B
175kDa-
1.0 0.8 4.1 2.7 1.3 0.5 1.0 0.6 1.3 0.5 0.2 0.1 1.0 0.6 3.7 0.9 0.5 1.8
P70 S6 Kinase
70kDa-
1.0 0.4 1.9 0.4 1.3 0.4 1.0 0.4 0.8 0.4 1.2 0.4 1.0 0.2 0.4 0.1 0.2 0.2
P44/42 MAPK
44/42kDa-
1.0 1.3 1.9 1.0 0.5 0.1 1.0 3.1 2.4 2.1 2.4 2.4 1.0 0.2 0.4 0.5 0.3 0.1
pAKT (S473)
70kDa-
 1.0 0.2 1.0 0.1 0.4 0.1 1.0 0.3 0.9 0.2 0.8 0. 2 1.0 0.3 0.8 0.3 0.7 0.1
37kDa-
GAPDH

## Slide 2
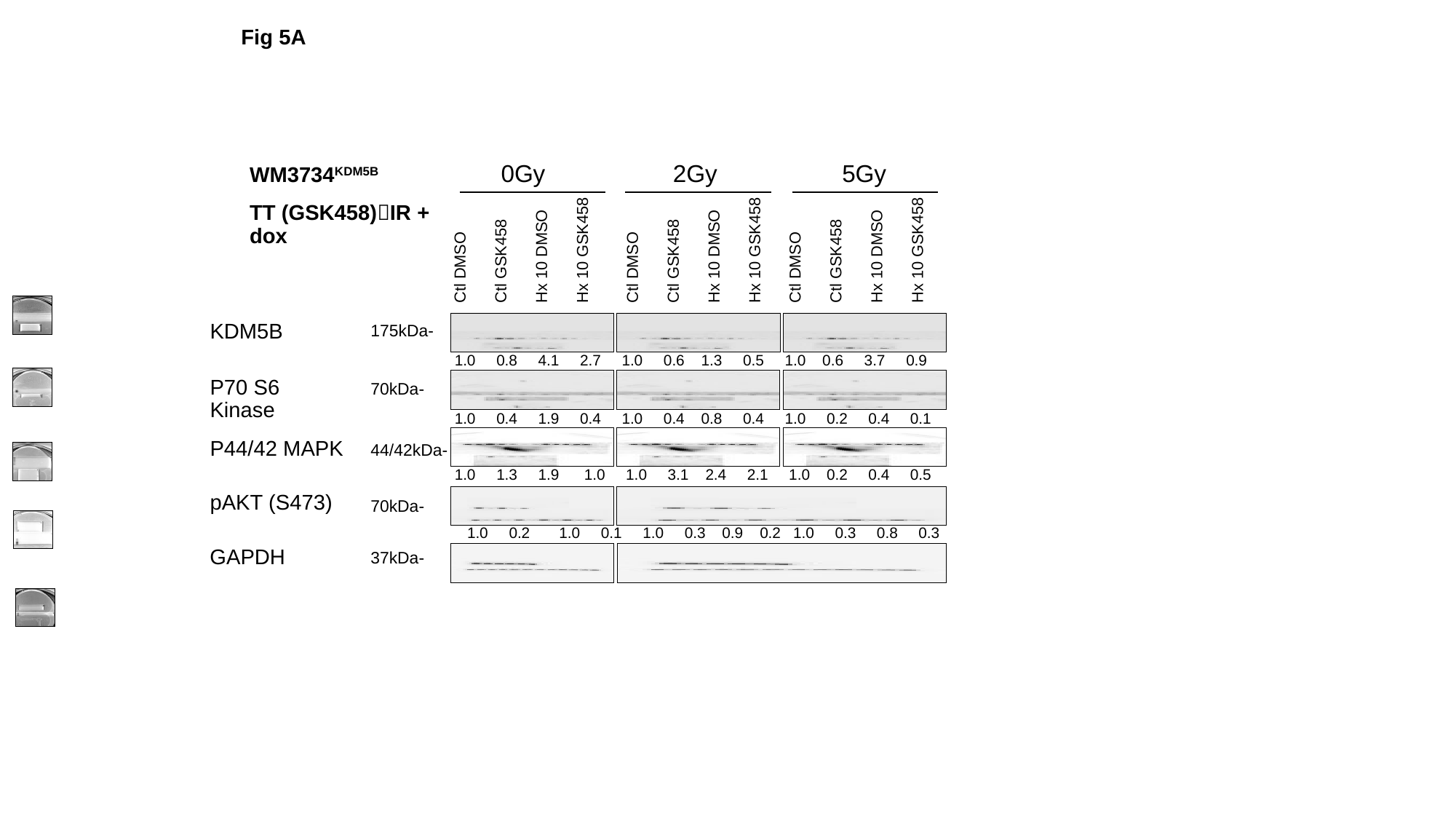

Fig 5A
0Gy
2Gy
5Gy
WM3734KDM5B
TT (GSK458)IR + dox
Hx 10 GSK458
Hx 10 GSK458
Hx 10 GSK458
Hx 10 DMSO
Hx 10 DMSO
Hx 10 DMSO
Ctl GSK458
Ctl GSK458
Ctl GSK458
Ctl DMSO
Ctl DMSO
Ctl DMSO
KDM5B
175kDa-
1.0 0.8 4.1 2.7 1.0 0.6 1.3 0.5 1.0 0.6 3.7 0.9
P70 S6 Kinase
70kDa-
1.0 0.4 1.9 0.4 1.0 0.4 0.8 0.4 1.0 0.2 0.4 0.1
P44/42 MAPK
44/42kDa-
1.0 1.3 1.9 1.0 1.0 3.1 2.4 2.1 1.0 0.2 0.4 0.5
pAKT (S473)
70kDa-
 1.0 0.2 1.0 0.1 1.0 0.3 0.9 0.2 1.0 0.3 0.8 0.3
37kDa-
GAPDH

## Slide 3
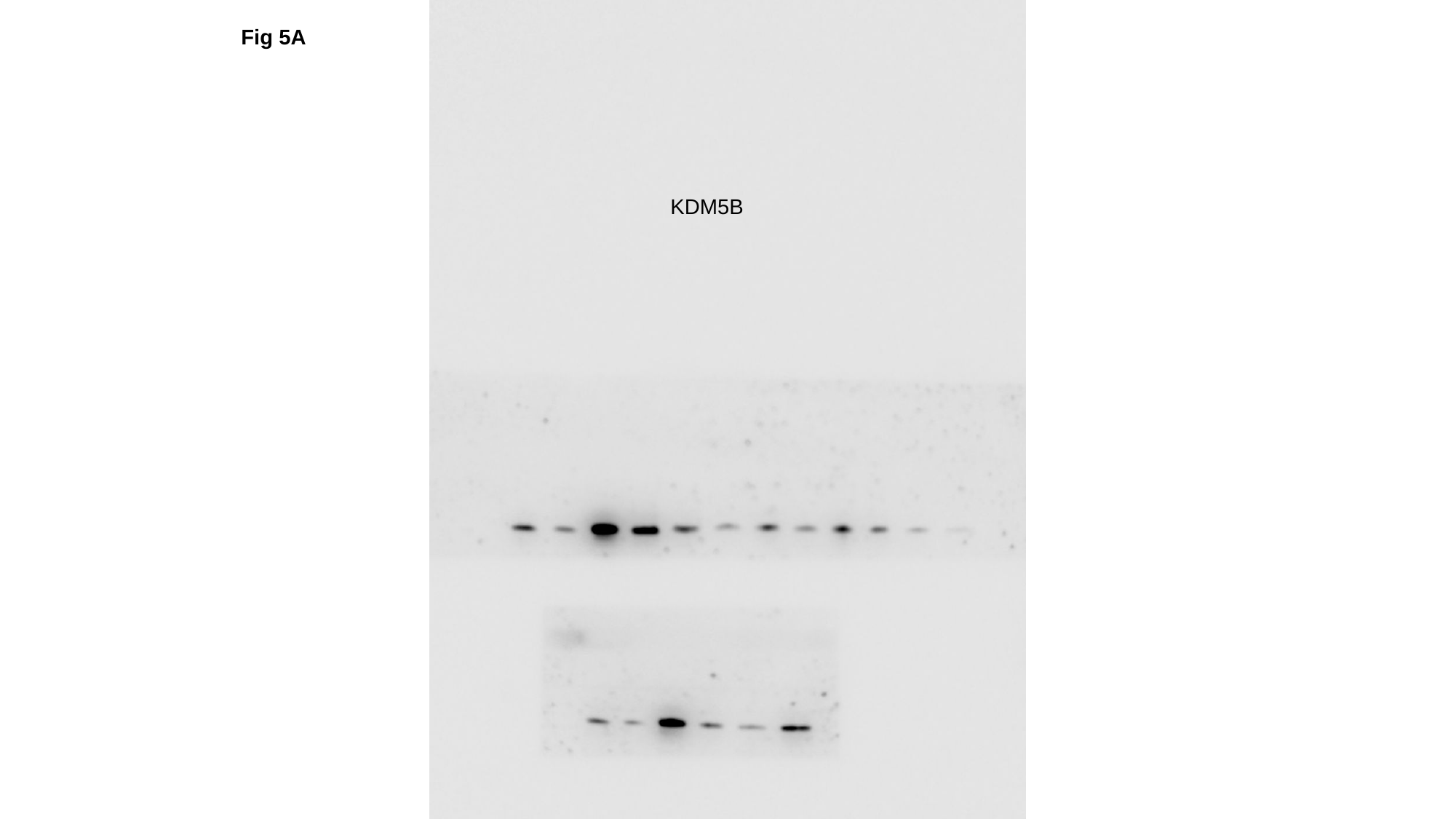

Fig 5A
KDM5B

## Slide 4
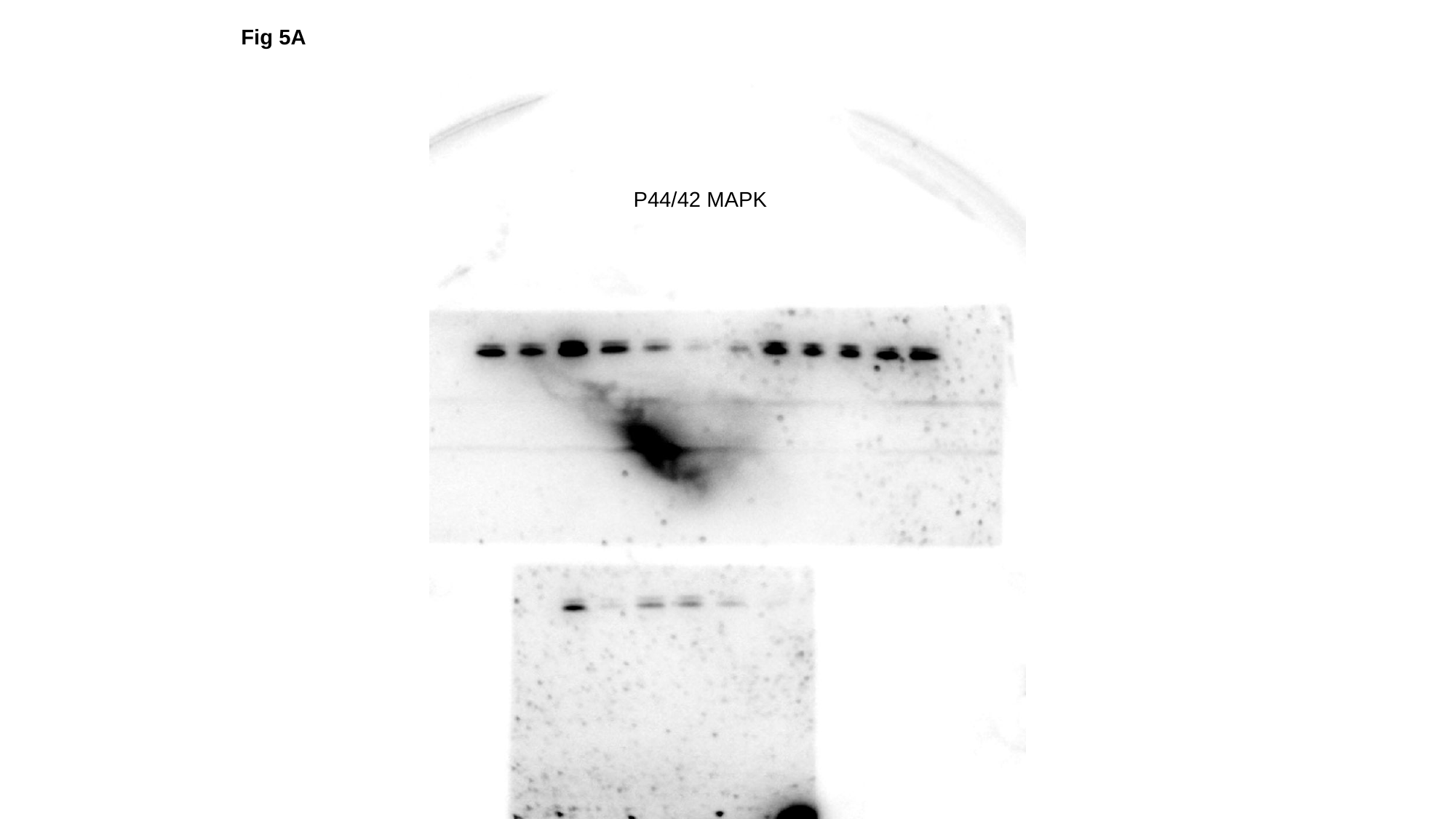

Fig 5A
P44/42 MAPK

## Slide 5
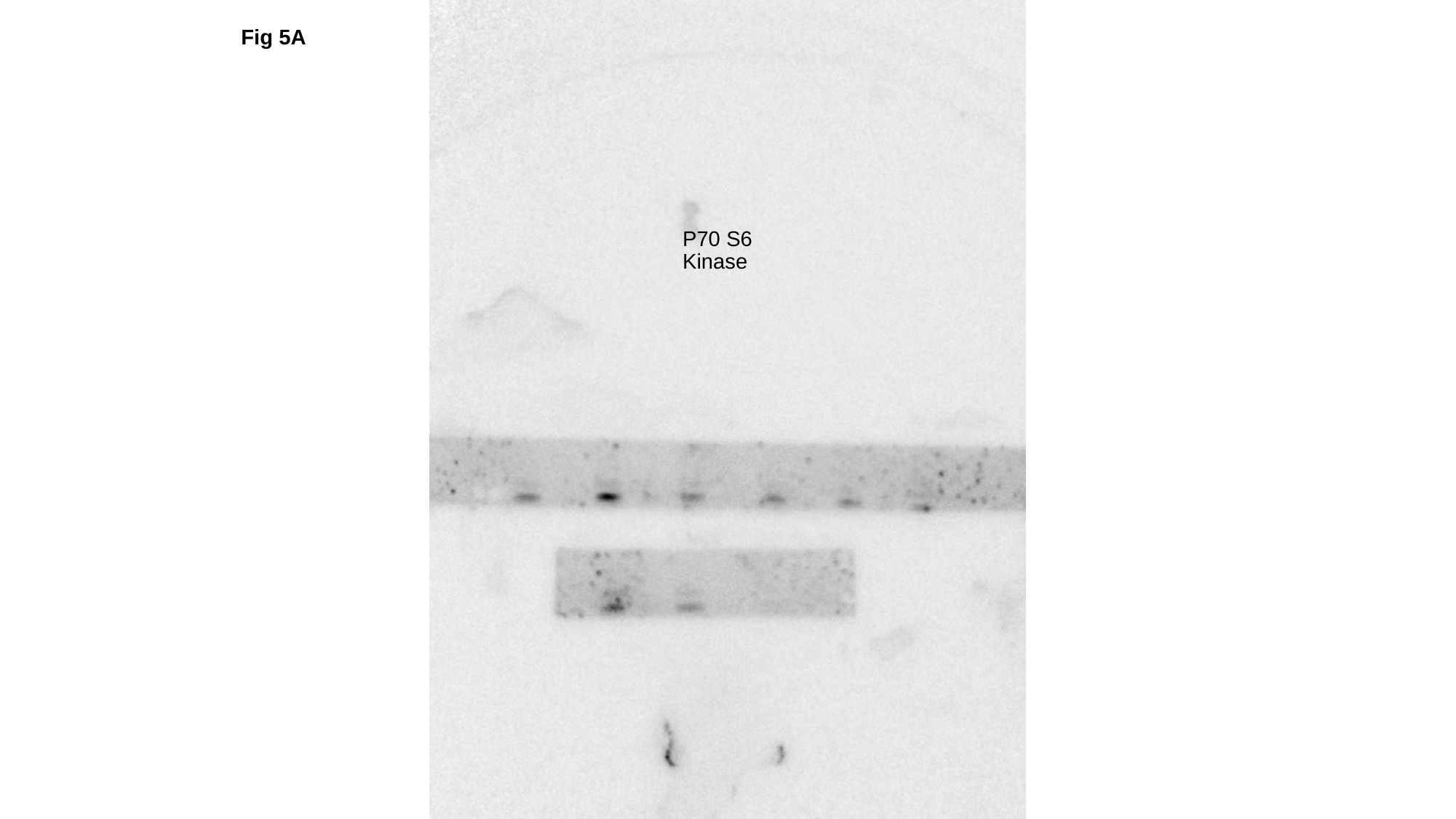

Fig 5A
P70 S6 Kinase

## Slide 6
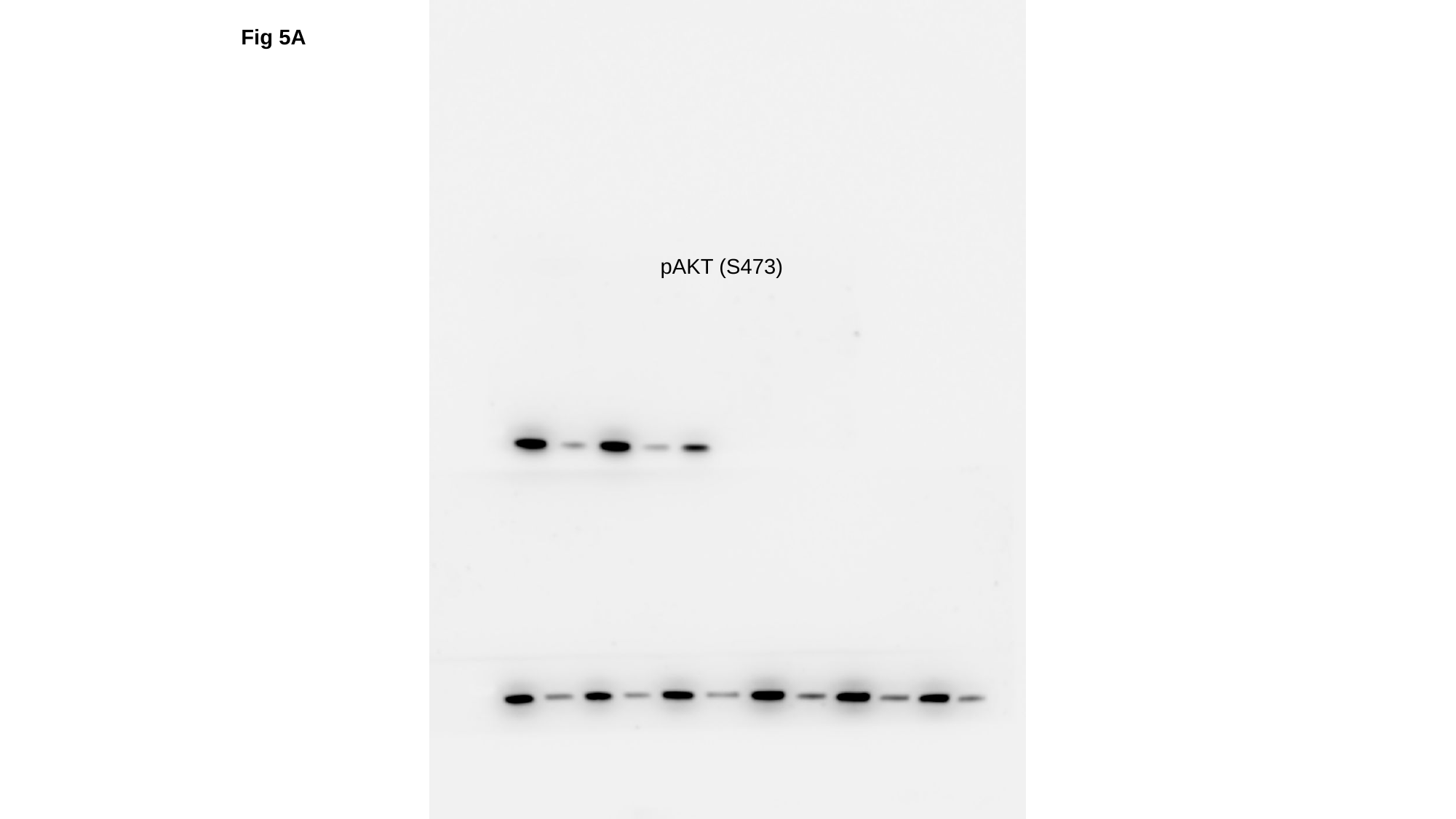

Fig 5A
pAKT (S473)

## Slide 7
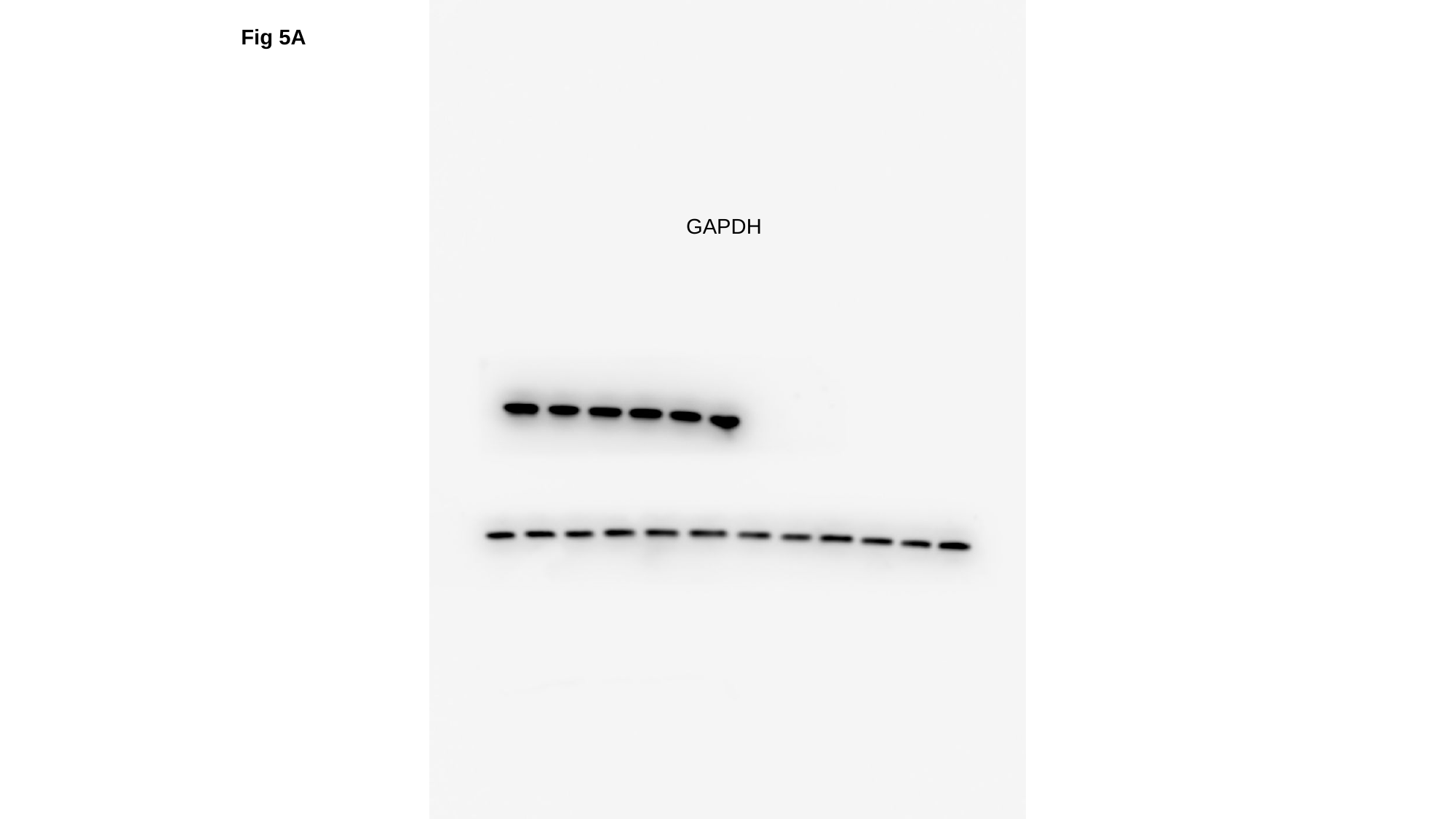

Fig 5A
GAPDH

## Slide 8
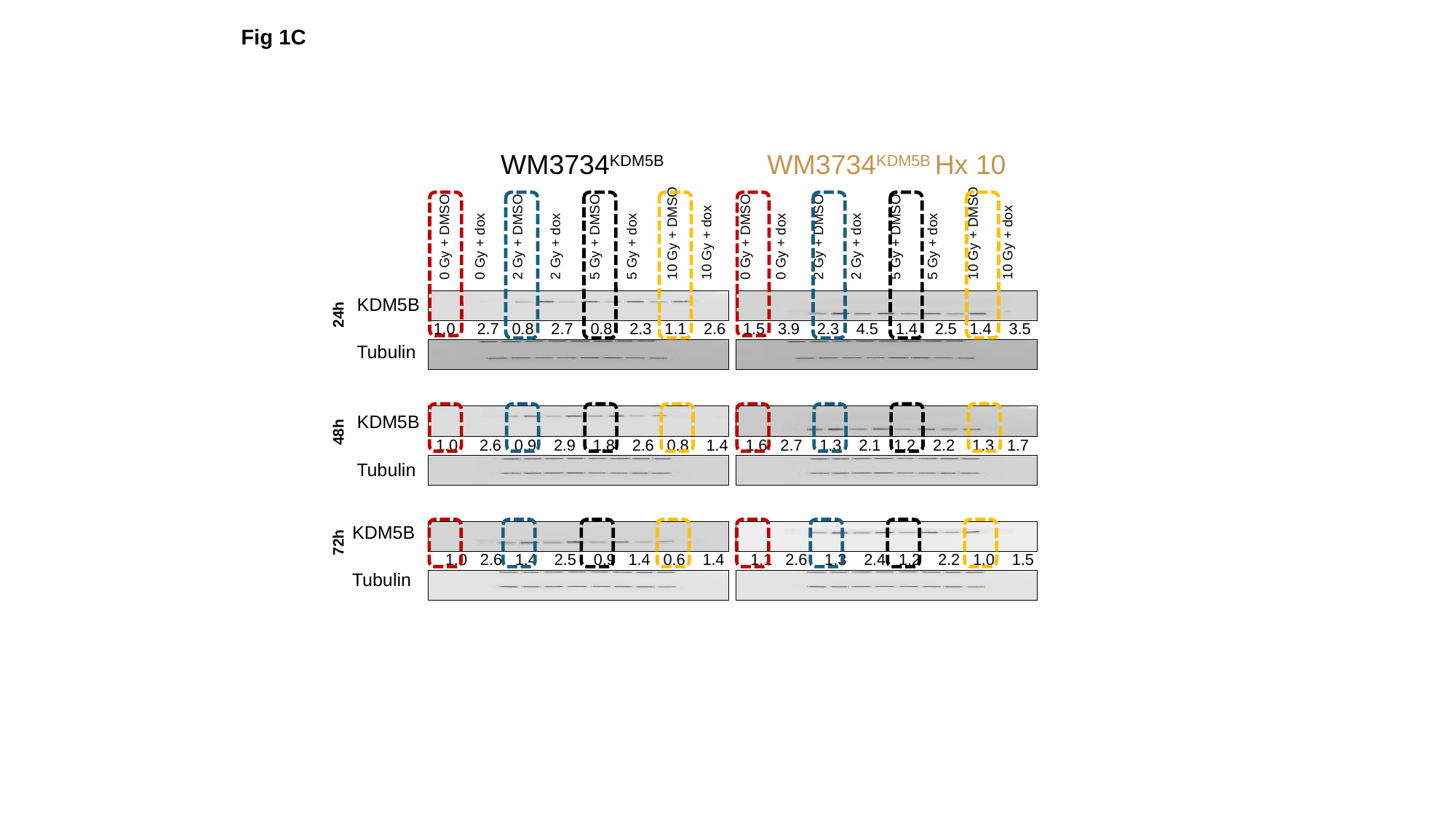

Fig 1C
WM3734KDM5B
WM3734KDM5B Hx 10
10 Gy + DMSO
10 Gy + DMSO
0 Gy + DMSO
2 Gy + DMSO
5 Gy + DMSO
0 Gy + DMSO
2 Gy + DMSO
5 Gy + DMSO
10 Gy + dox
10 Gy + dox
0 Gy + dox
2 Gy + dox
5 Gy + dox
0 Gy + dox
2 Gy + dox
5 Gy + dox
KDM5B
24h
 1.0 2.7 0.8 2.7 0.8 2.3 1.1 2.6 1.5 3.9 2.3 4.5 1.4 2.5 1.4 3.5
Tubulin
KDM5B
48h
 1.0 2.6 0.9 2.9 1.8 2.6 0.8 1.4 1.6 2.7 1.3 2.1 1.2 2.2 1.3 1.7
Tubulin
KDM5B
72h
 1.0 2.6 1.4 2.5 0.9 1.4 0.6 1.4 1.1 2.6 1.3 2.4 1.2 2.2 1.0 1.5
Tubulin

## Slide 9
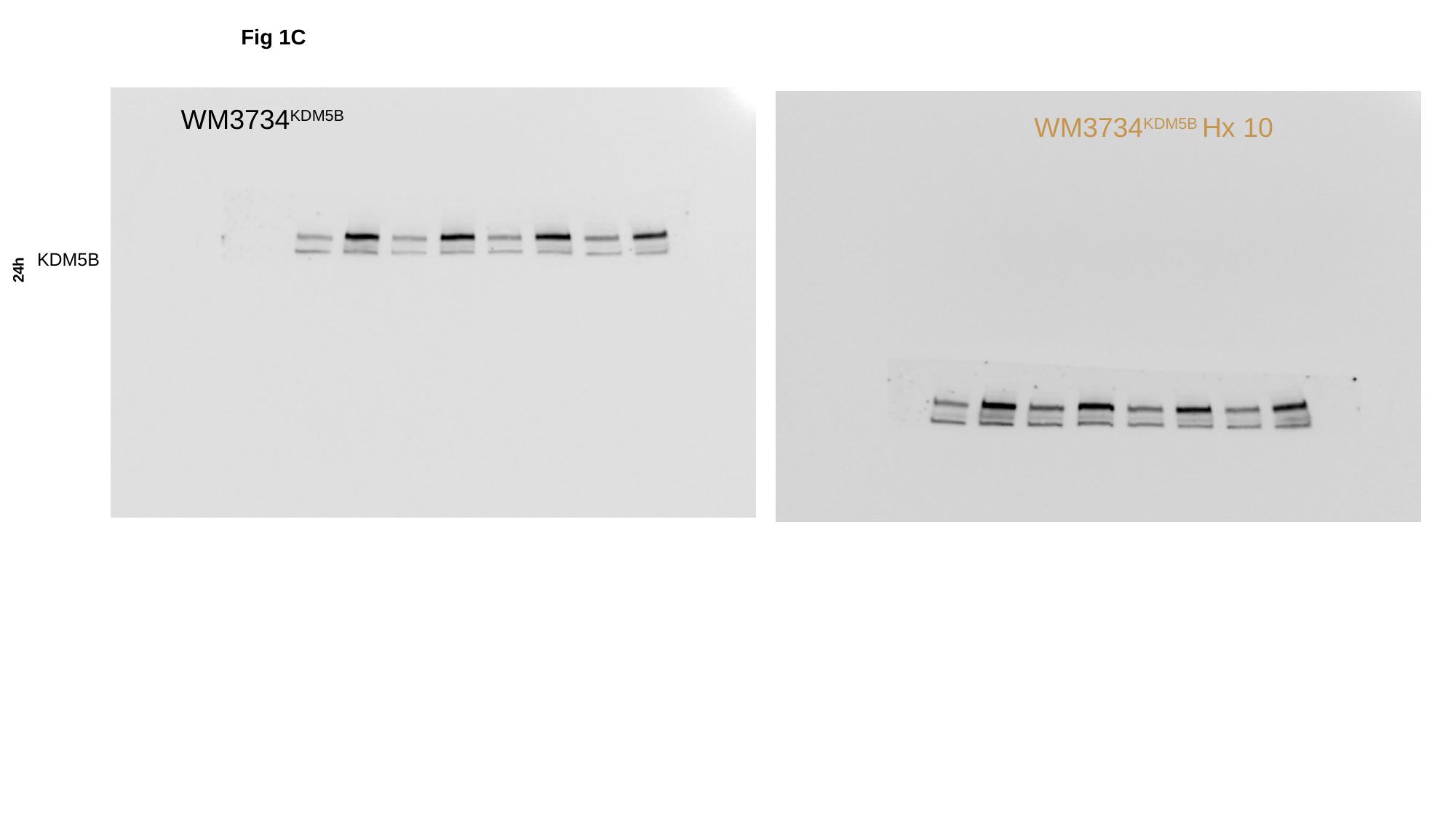

Fig 1C
WM3734KDM5B
WM3734KDM5B Hx 10
KDM5B
24h

## Slide 10
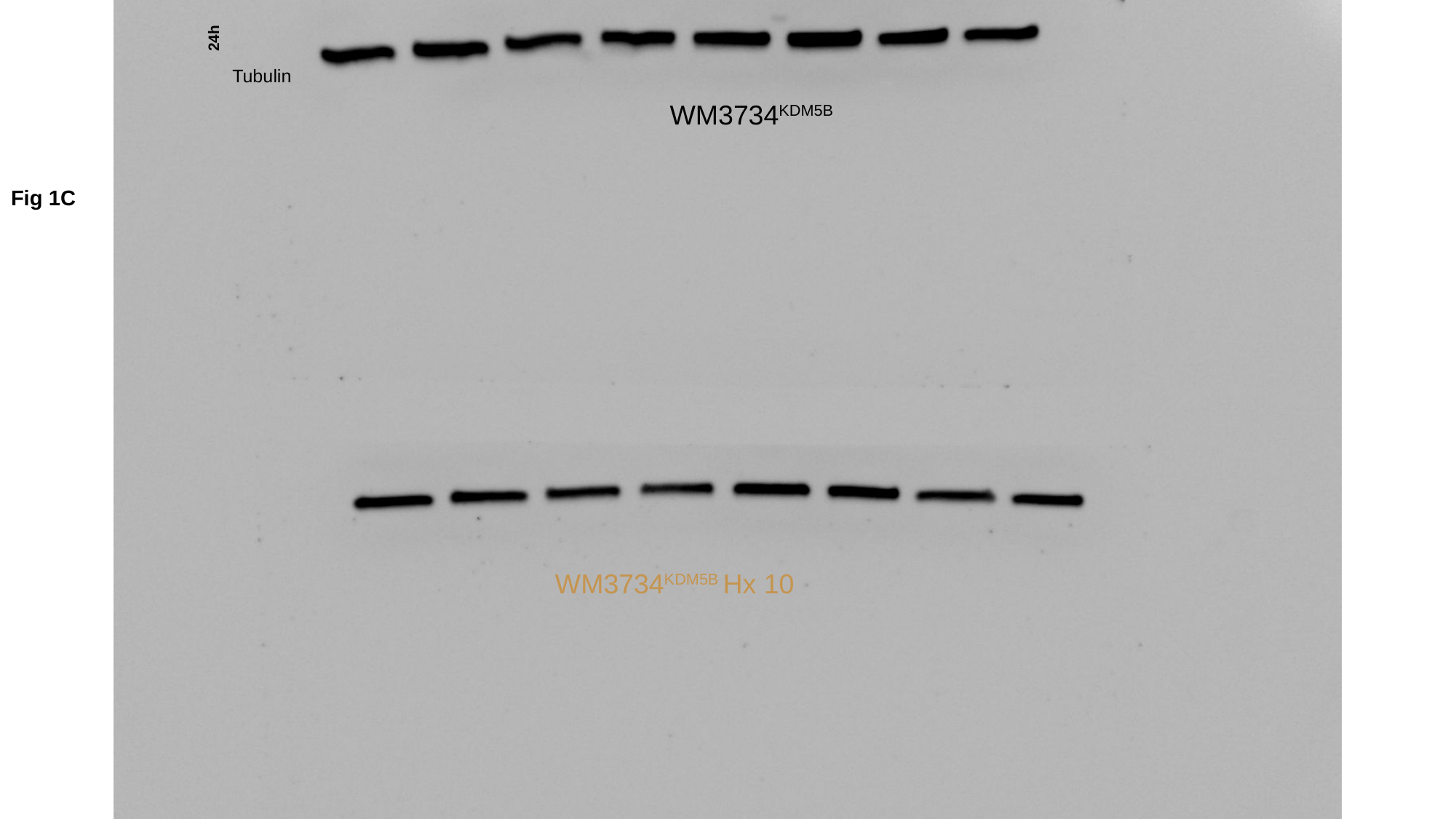

24h
Tubulin
WM3734KDM5B
Fig 1C
WM3734KDM5B Hx 10

## Slide 11
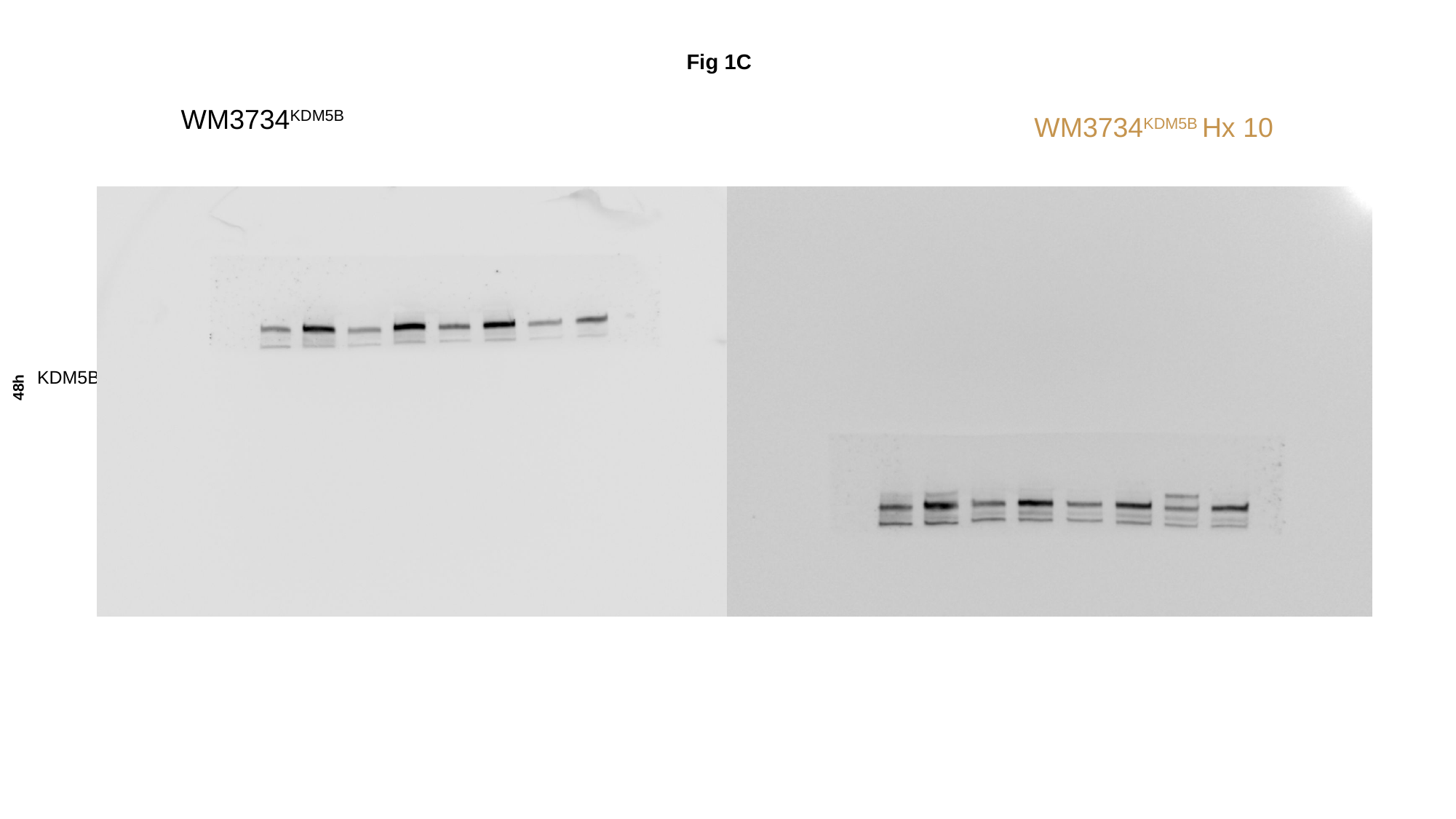

Fig 1C
WM3734KDM5B
WM3734KDM5B Hx 10
KDM5B
48h

## Slide 12
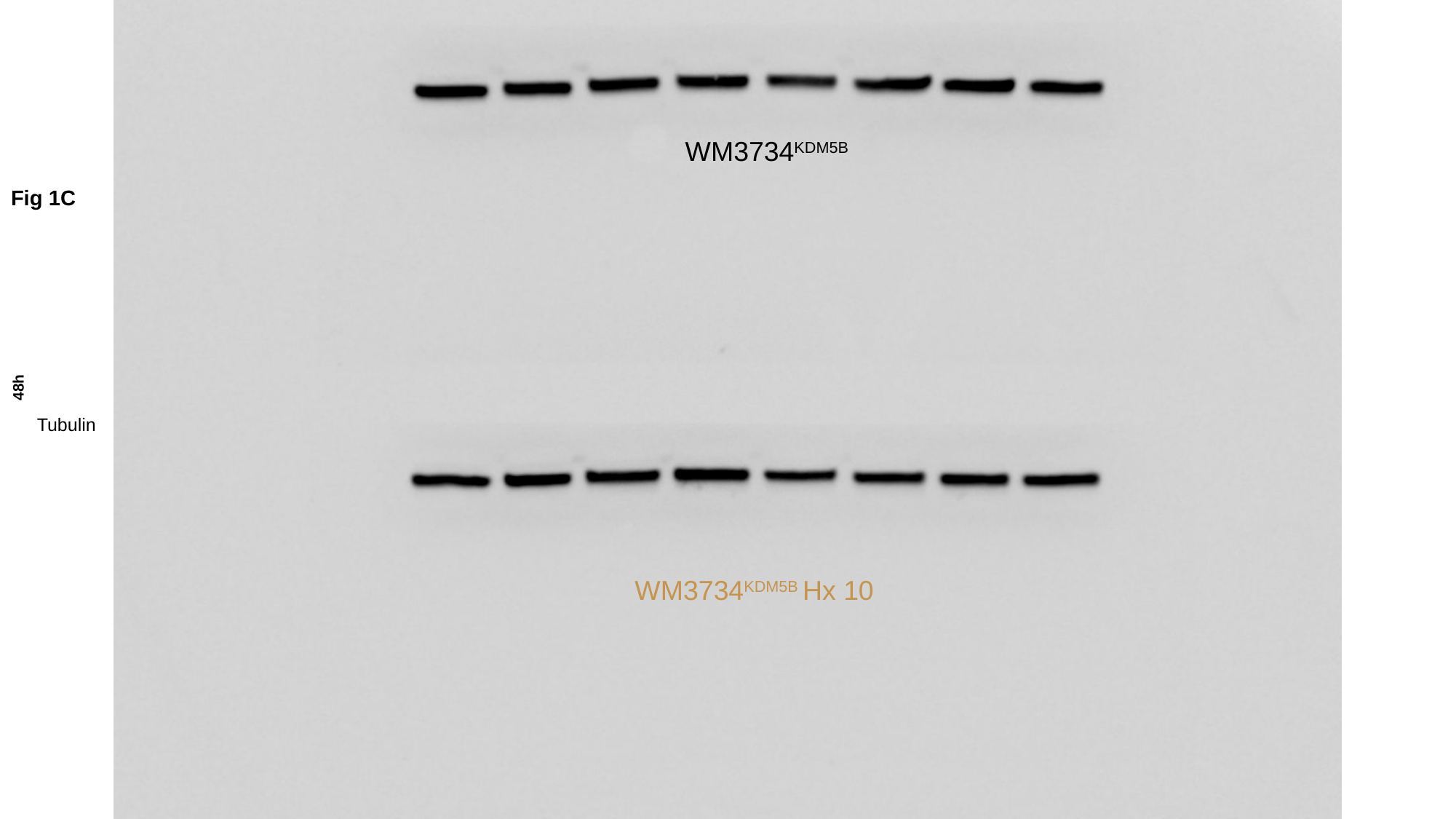

WM3734KDM5B
Fig 1C
48h
Tubulin
WM3734KDM5B Hx 10

## Slide 13
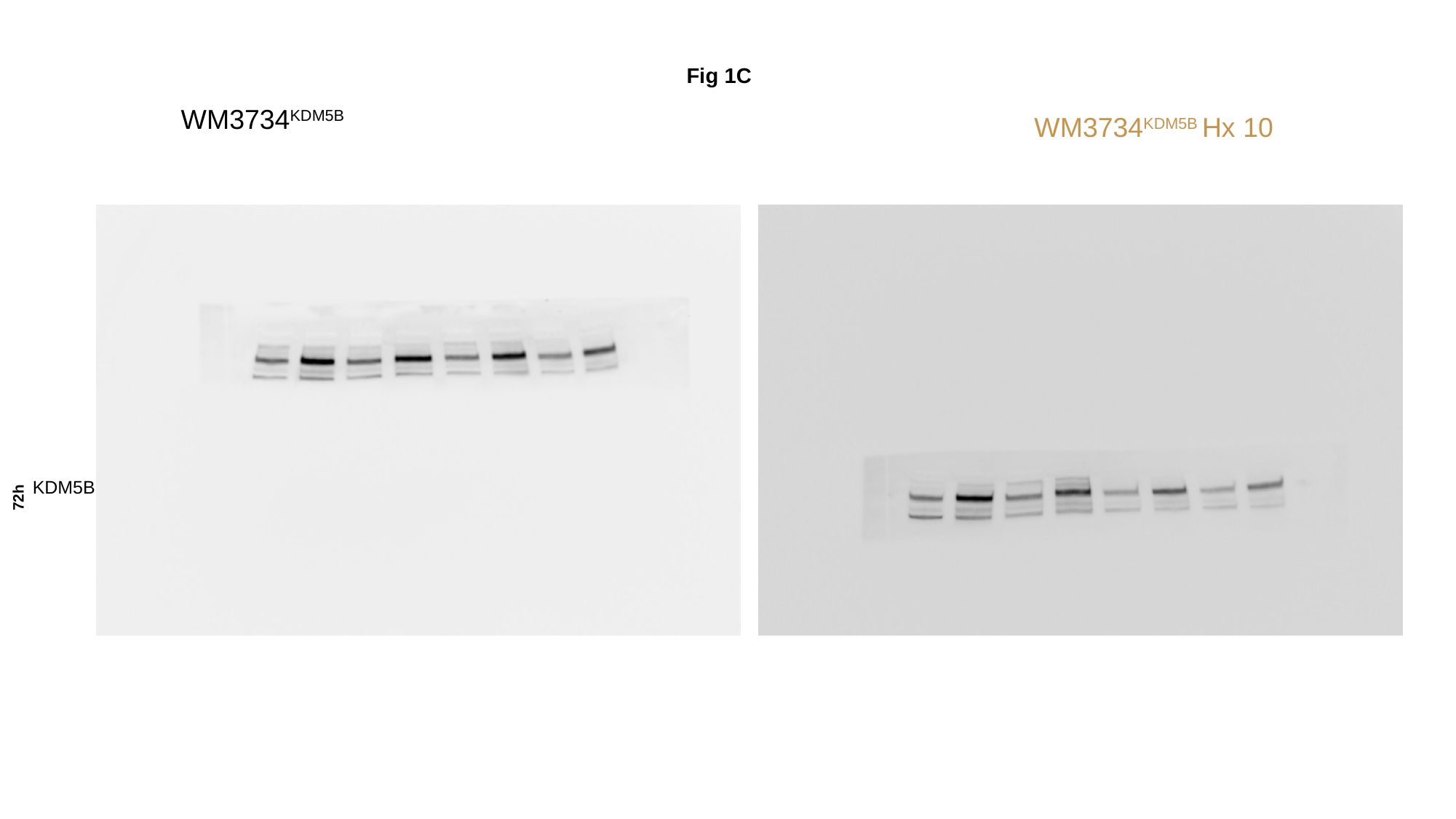

Fig 1C
WM3734KDM5B
WM3734KDM5B Hx 10
KDM5B
72h

## Slide 14
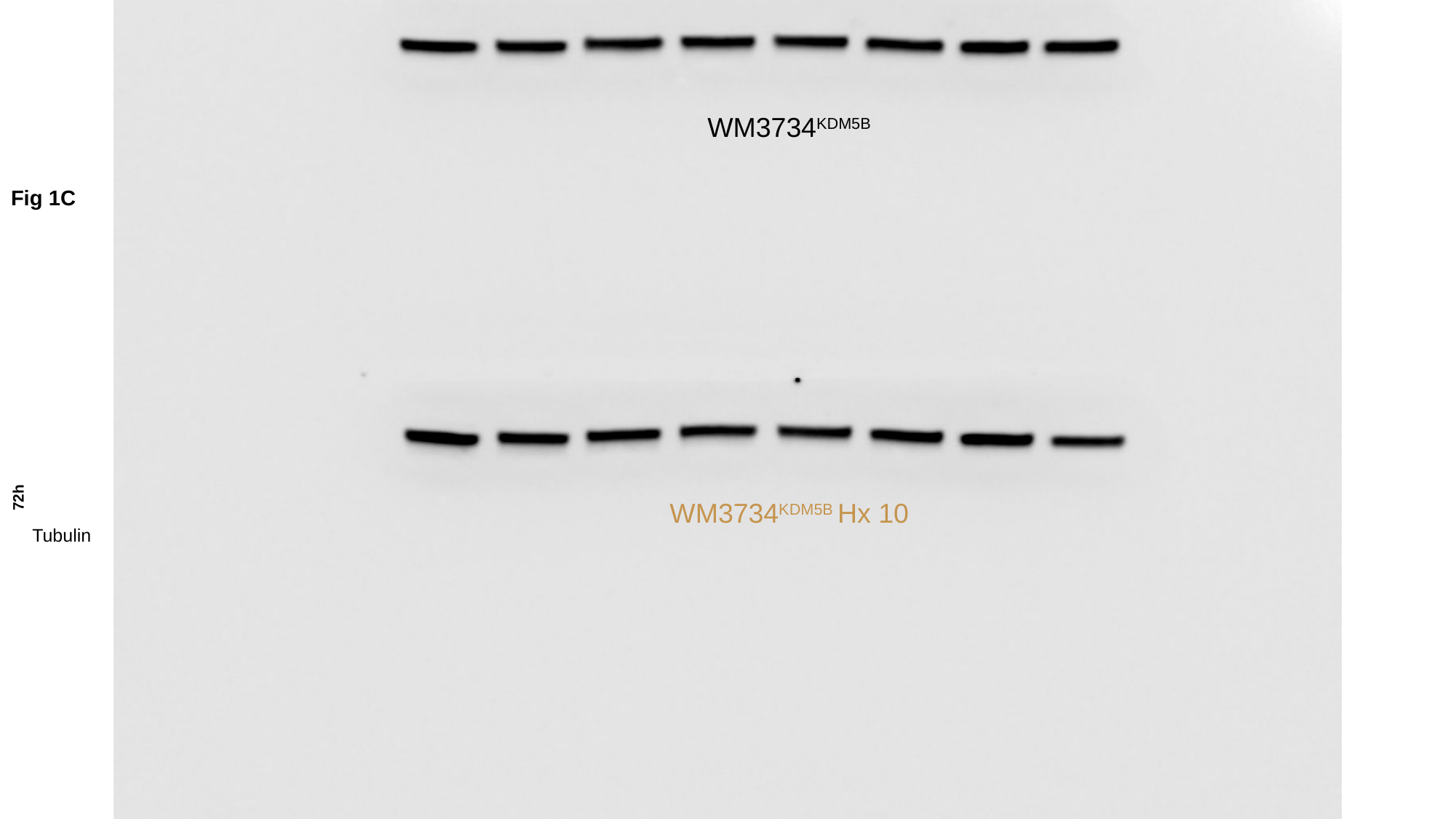

WM3734KDM5B
Fig 1C
72h
WM3734KDM5B Hx 10
Tubulin

## Slide 15
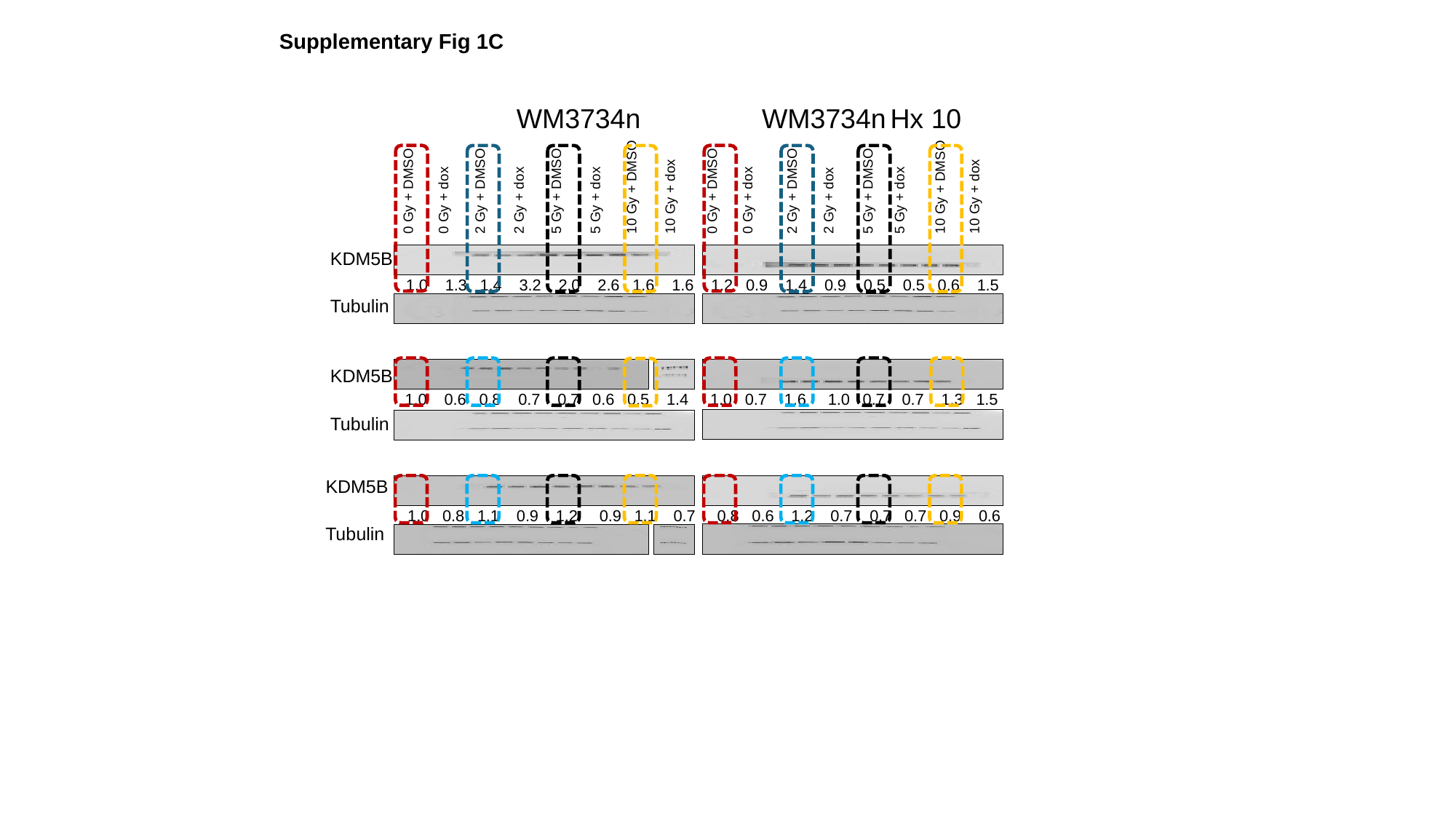

Supplementary Fig 1C
WM3734n
WM3734n Hx 10
10 Gy + DMSO
10 Gy + DMSO
0 Gy + DMSO
2 Gy + DMSO
5 Gy + DMSO
0 Gy + DMSO
2 Gy + DMSO
5 Gy + DMSO
10 Gy + dox
10 Gy + dox
0 Gy + dox
2 Gy + dox
5 Gy + dox
0 Gy + dox
2 Gy + dox
5 Gy + dox
KDM5B
 1.0 1.3 1.4 3.2 2.0 2.6 1.6 1.6 1.2 0.9 1.4 0.9 0.5 0.5 0.6 1.5
Tubulin
KDM5B
 1.0 0.6 0.8 0.7 0.7 0.6 0.5 1.4 1.0 0.7 1.6 1.0 0.7 0.7 1.3 1.5
Tubulin
KDM5B
 1.0 0.8 1.1 0.9 1.2 0.9 1.1 0.7 0.8 0.6 1.2 0.7 0.7 0.7 0.9 0.6
Tubulin

## Slide 16
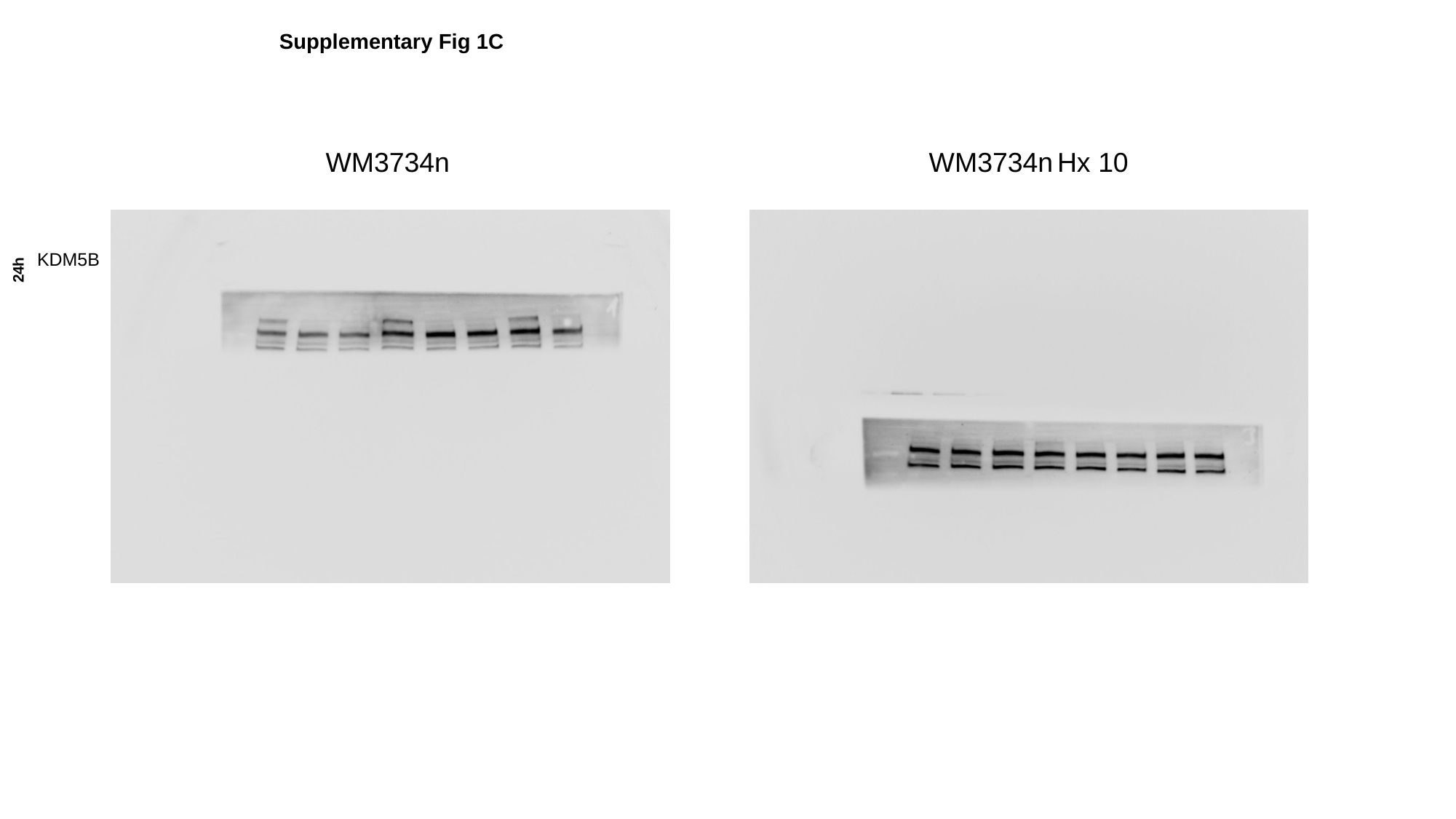

Supplementary Fig 1C
WM3734n
WM3734n Hx 10
KDM5B
24h

## Slide 17
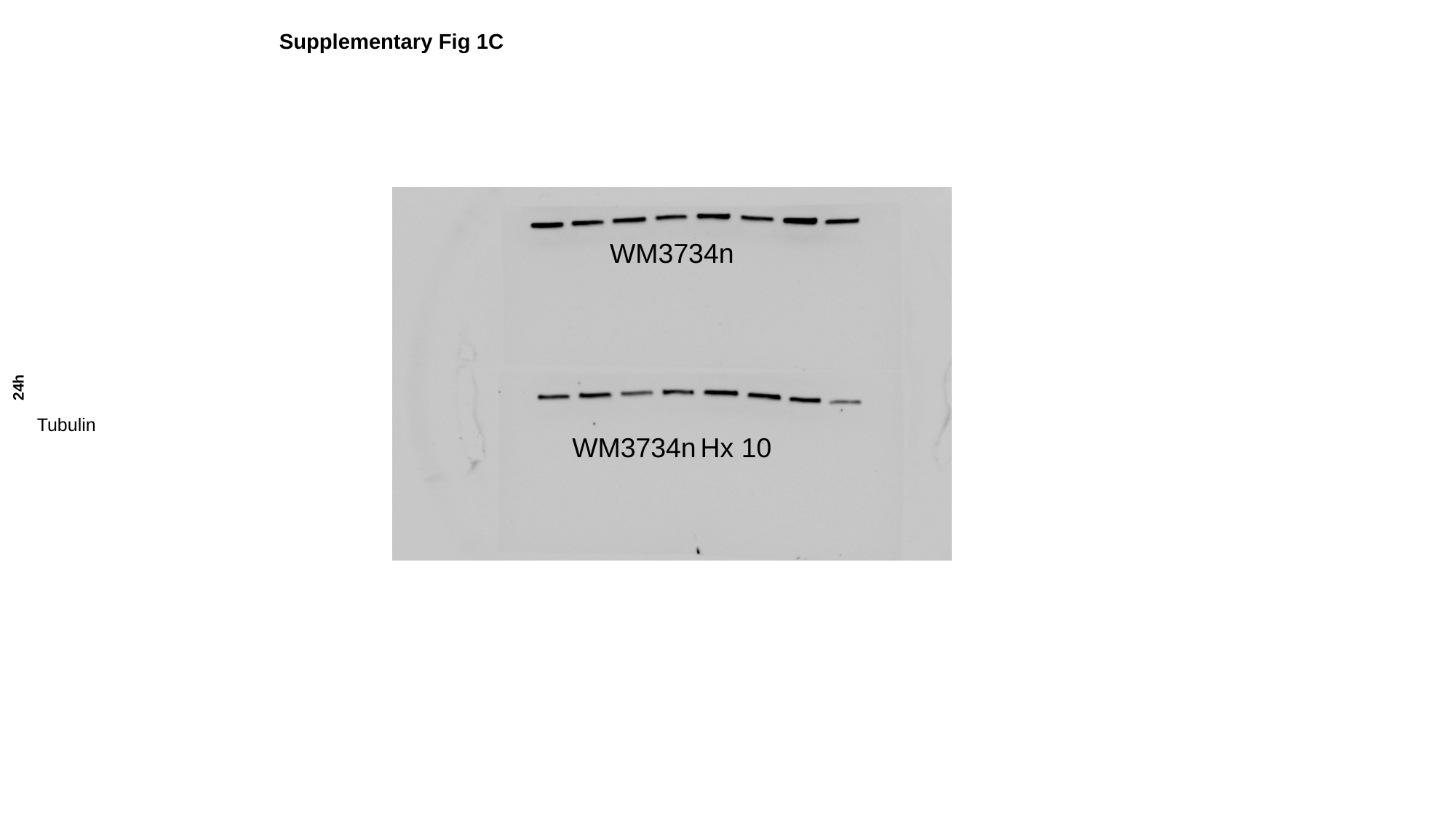

Supplementary Fig 1C
WM3734n
24h
Tubulin
WM3734n Hx 10

## Slide 18
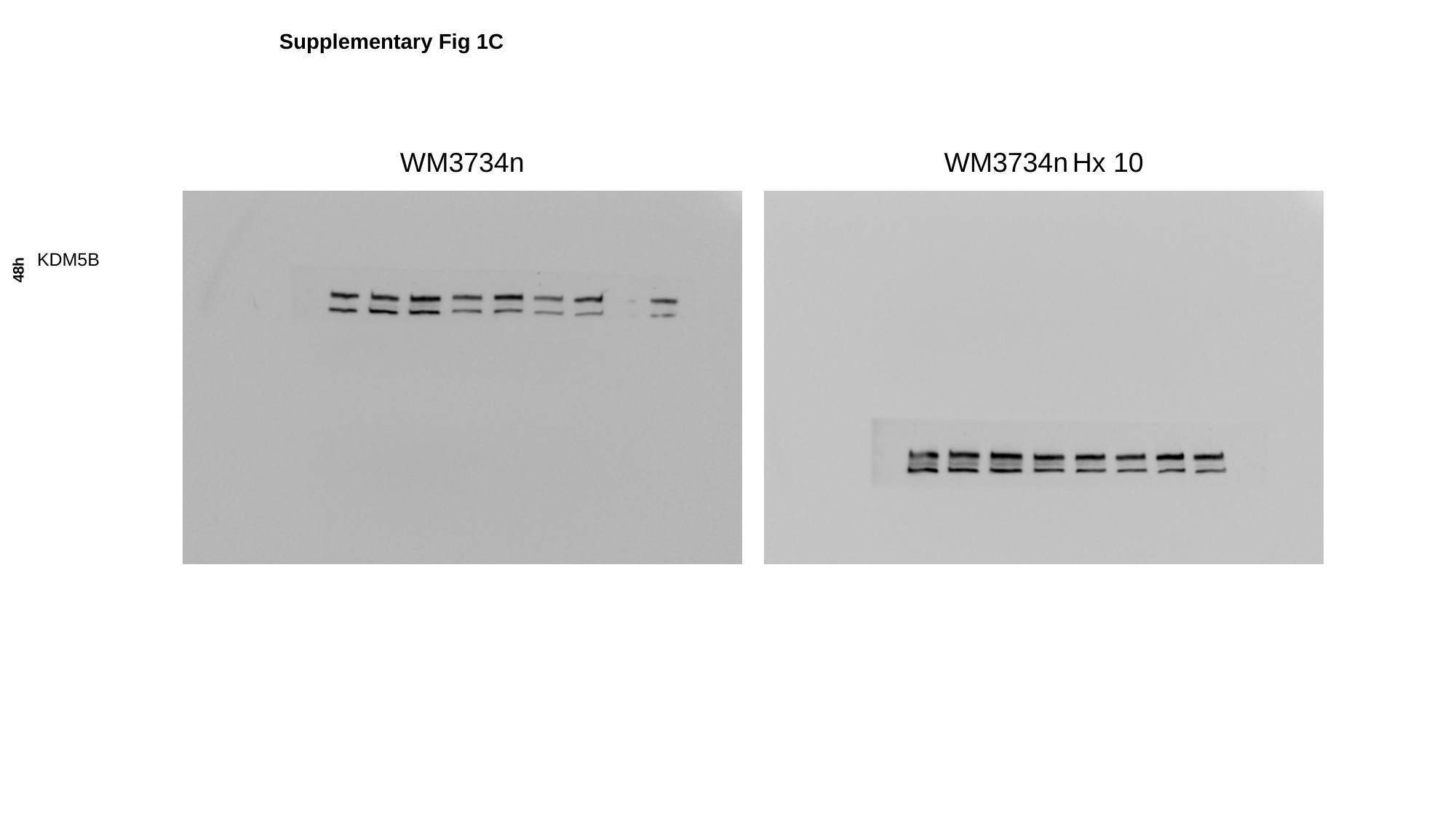

Supplementary Fig 1C
WM3734n
WM3734n Hx 10
KDM5B
48h

## Slide 19
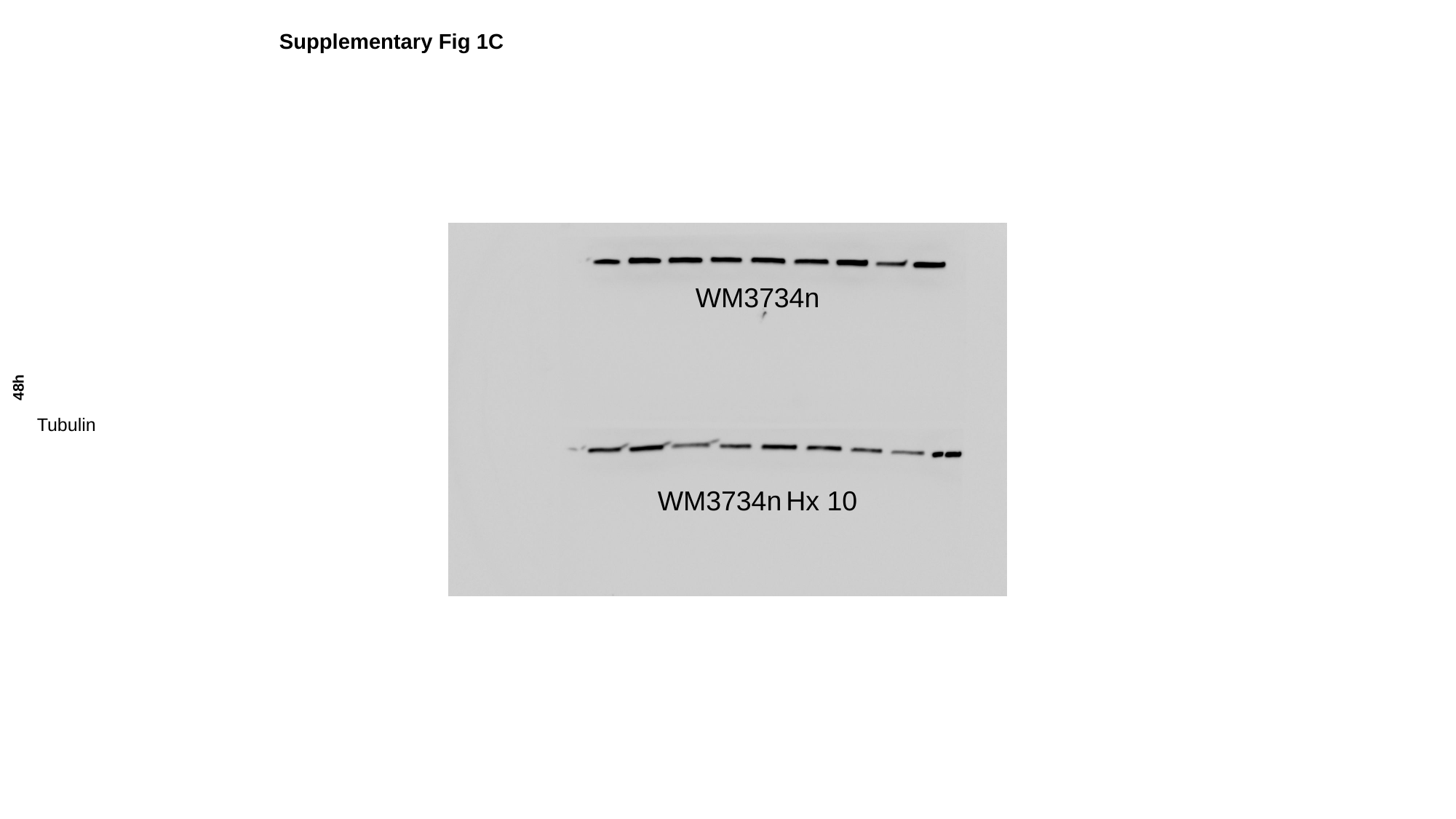

Supplementary Fig 1C
WM3734n
48h
Tubulin
WM3734n Hx 10

## Slide 20
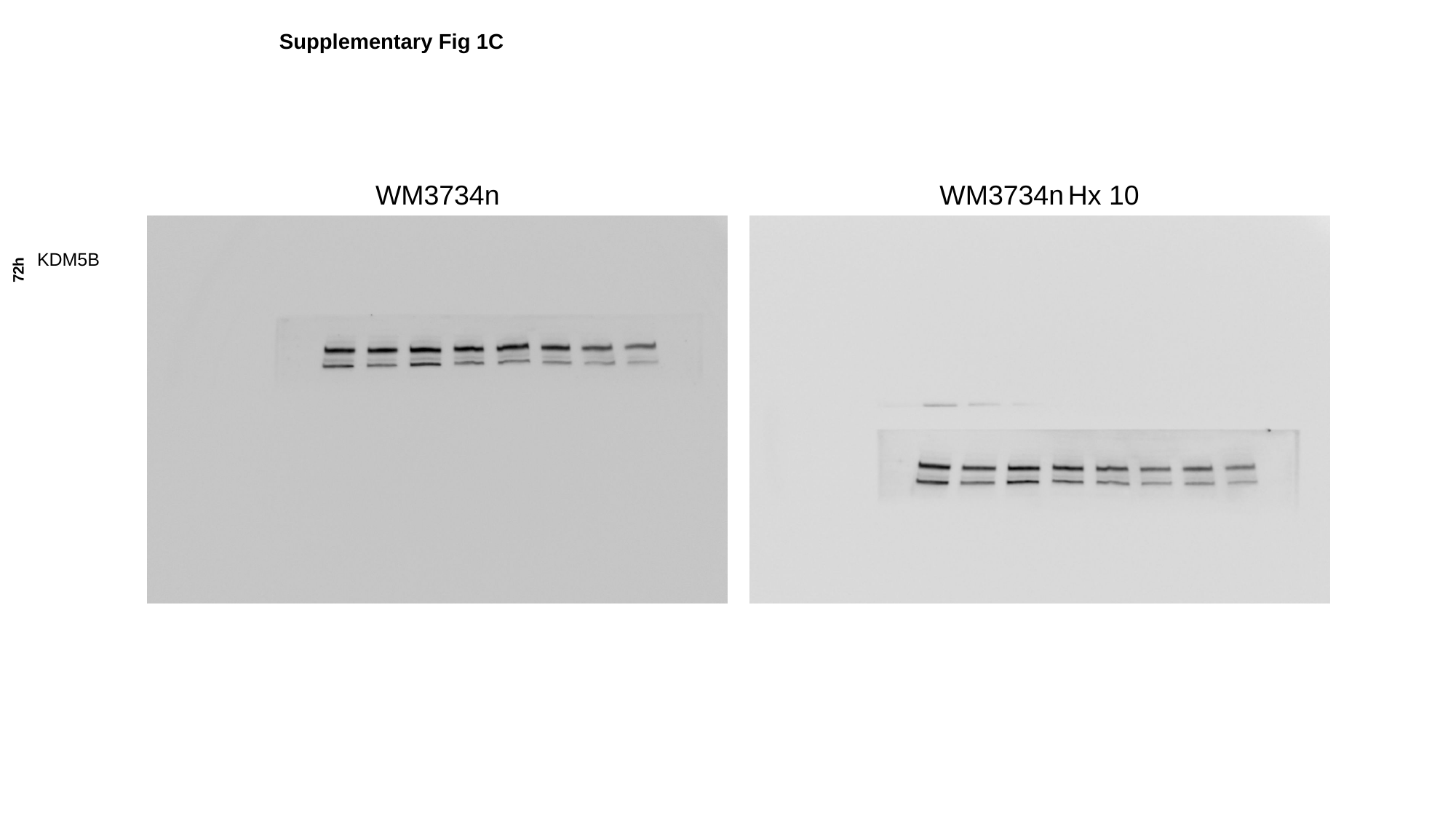

Supplementary Fig 1C
WM3734n
WM3734n Hx 10
KDM5B
72h

## Slide 21
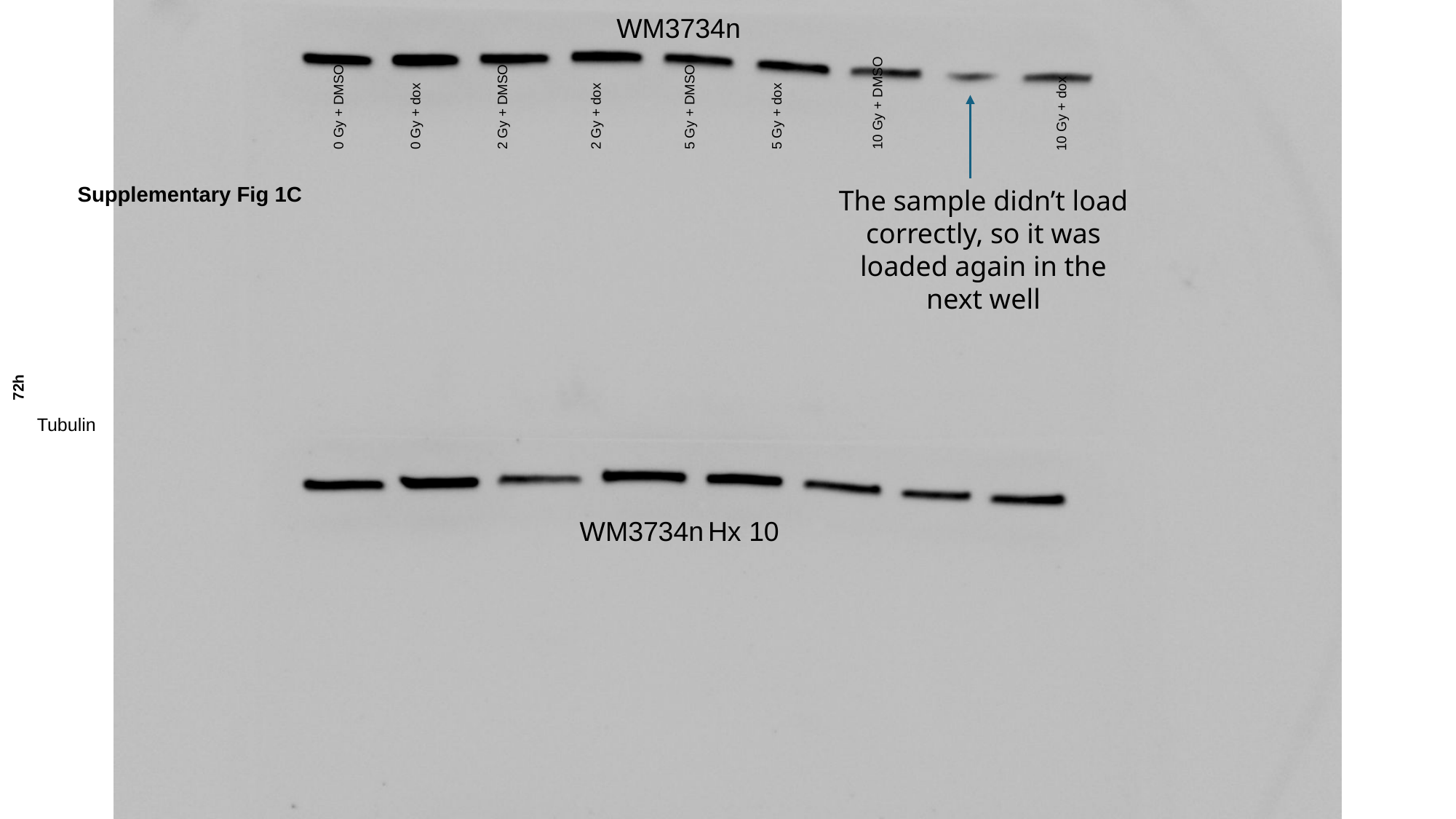

WM3734n
10 Gy + DMSO
0 Gy + DMSO
2 Gy + DMSO
5 Gy + DMSO
10 Gy + dox
0 Gy + dox
2 Gy + dox
5 Gy + dox
Supplementary Fig 1C
The sample didn’t load correctly, so it was loaded again in the next well
72h
Tubulin
WM3734n Hx 10

## Slide 22
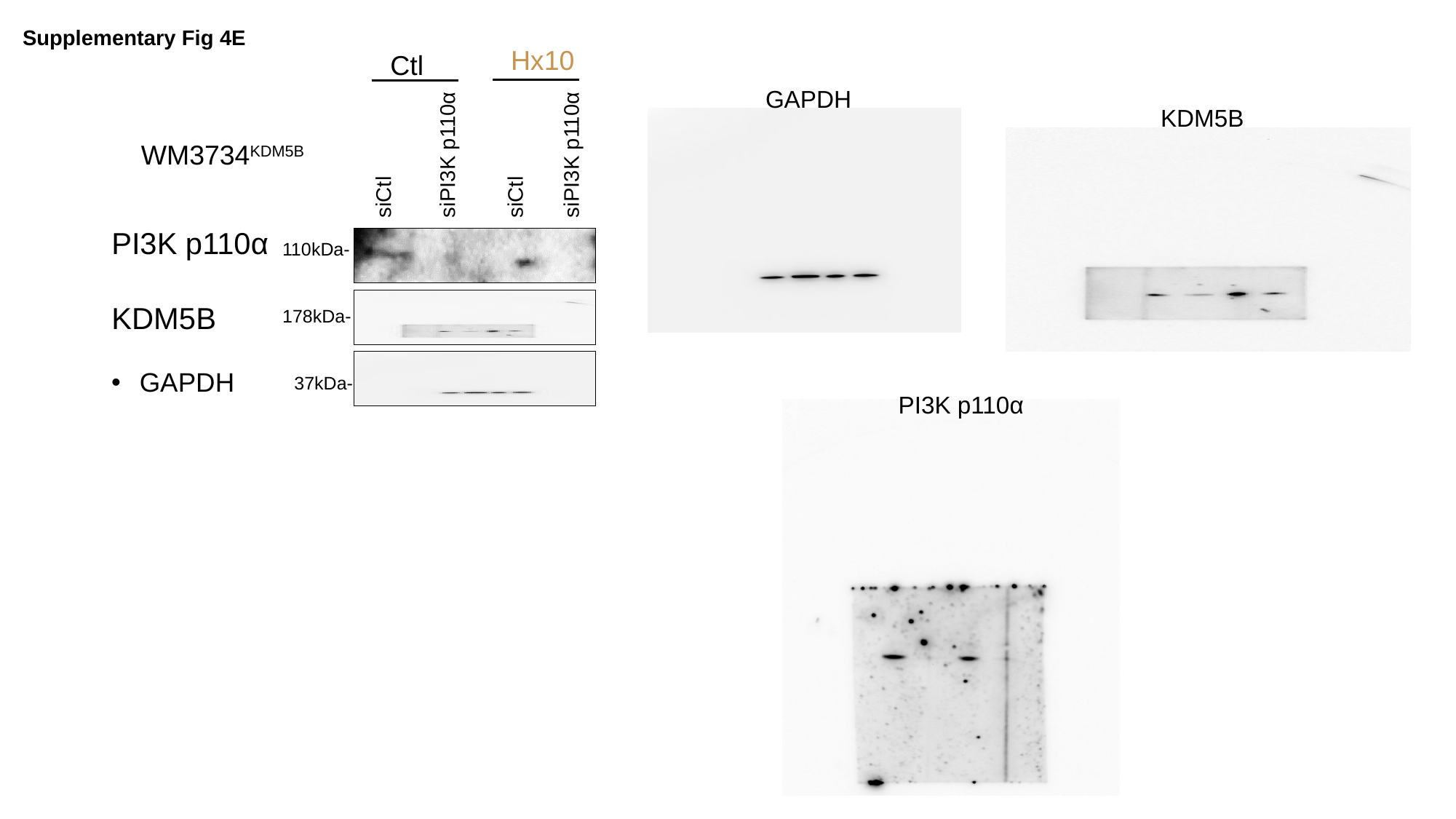

Supplementary Fig 4E
Hx10
Ctl
GAPDH
KDM5B
WM3734KDM5B
siPI3K p110α
siPI3K p110α
siCtl
siCtl
PI3K p110α
110kDa-
KDM5B
178kDa-
GAPDH
37kDa-
PI3K p110α
